# Cyclobutanone Mimics of Intermediates in Metallo‐β‐Lactamase Catalysis

**DOI:** 10.1002/chem.201705886

**Published:** 2018-01-17

**Authors:** Martine I. Abboud, Magda Kosmopoulou, Anthony P. Krismanich, Jarrod W. Johnson, Philip Hinchliffe, Jürgen Brem, Timothy D. W. Claridge, James Spencer, Christopher J. Schofield, Gary I. Dmitrienko

**Affiliations:** ^1^ Department of Chemistry University of Oxford 12 Mansfield Road Oxford OX1 3TA UK; ^2^ School of Cellular and Molecular Medicine University of Bristol, Medical Sciences Building Bristol BS8 1TD UK; ^3^ Department of Chemistry University of Waterloo 200 University Ave. W. Waterloo, Ontario N2L 3G1 Canada

**Keywords:** antimicrobial resistance, β-lactam analogues, β-lactamases, cyclobutanones, transition state analogues

## Abstract

The most important resistance mechanism to β‐lactam antibiotics involves hydrolysis by two β‐lactamase categories: the nucleophilic serine and the metallo‐β‐lactamases (SBLs and MBLs, respectively). Cyclobutanones are hydrolytically stable β‐lactam analogues with potential to inhibit both SBLs and MBLs. We describe solution and crystallographic studies on the interaction of a cyclobutanone penem analogue with the clinically important MBL SPM‐1. NMR experiments using ^19^F‐labeled SPM‐1 imply the cyclobutanone binds to SPM‐1 with micromolar affinity. A crystal structure of the SPM‐1:cyclobutanone complex reveals binding of the hydrated cyclobutanone through interactions with one of the zinc ions, stabilisation of the hydrate by hydrogen bonding to zinc‐bound water, and hydrophobic contacts with aromatic residues. NMR analyses using a ^13^C‐labeled cyclobutanone support assignment of the bound species as the hydrated ketone. The results inform on how MBLs bind substrates and stabilize tetrahedral intermediates. They support further investigations on the use of transition‐state and/or intermediate analogues as inhibitors of all β‐lactamase classes.

For more than 70 years, the β‐lactams (Figure 1) have been the most widely used antibacterials. β‐Lactam resistance is endemic, substantially due to β‐lactamases, which have two mechanistic classes—the nucleophilic serine β‐lactamases (SBLs) and the zinc‐dependent metallo‐β‐lactamases (MBLs) (Figure S1 in the Supporting Information).^1^ Combinations of a β‐lactam and a β‐lactam containing SBL inhibitor (e.g., clavulanic acid) have been clinically effective;^2^ however, the growing dissemination of new SBLs and MBLs, which are unaffected by such inhibitors, compromises this approach.^3^ Carbapenems are broad‐spectrum antibacterials, which were once often used as last‐line treatments. Their widespread use has led to the spread of SBL and MBL carbapenemases, especially in Gram‐negative bacteria, for example, *Escherichia coli* and *Klebsiella pneumoniae*.^4^ Examples include both Class A and D SBLs and Class B MBLs (e.g., IMP‐1, VIM‐2, SPM‐1, NDM‐1). Avibactam has been introduced as a broad‐spectrum SBL inhibitor and is the first clinically useful non‐β‐lactam β‐lactamase inhibitor;^5^ however, it is a (poor) substrate of some SBLs and most MBLs.^6^ There is thus an unmet need for hydrolytically stable inhibitors active against both SBLs and MBLs.

**Figure 1:**
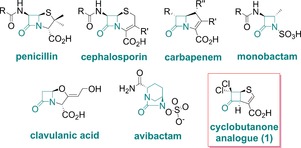
Structures of major classes of clinically used β‐lactams, serine β‐lactamase inhibitors, cyclobutanone analogue (**1**), and avibactam.

One approach to obtain inhibitors active against the two mechanistically distinct classes of β‐lactamases is to mimic the common tetrahedral intermediate (Figure 2 A) or transition states pre‐ or succeeding it.^7^ Although increasing numbers of structures describe binding of hydrolyzed β‐lactams to MBLs, progress in inhibitor development is hampered by the absence of structures describing interactions of MBLs with intact substrates/close analogues. We, and others, have been exploring cyclobutanone analogues of β‐lactams as mechanistic probes and as templates for broad spectrum β‐lactamase inhibition (Figure 2 B). Early compounds, however, manifested only weak Class A SBL inhibition.^8^ Recently, we have found that cyclobutanone analogues of the penems and penams inhibit both SBLs and MBLs.8a We identified the cyclobutanone penem analogue **1** (Figure 1) to be the most potent compound tested against class A and C SBLs, and to have modest inhibition of the IMP‐1 MBL.8a However, although we obtained crystallographic evidence for SBL inhibition, involving binding of the cyclobutanone by a hemiketal to the nucleophilic serine,8a no information has been available on how cyclobutanones inhibit MBLs.

**Figure 2:**
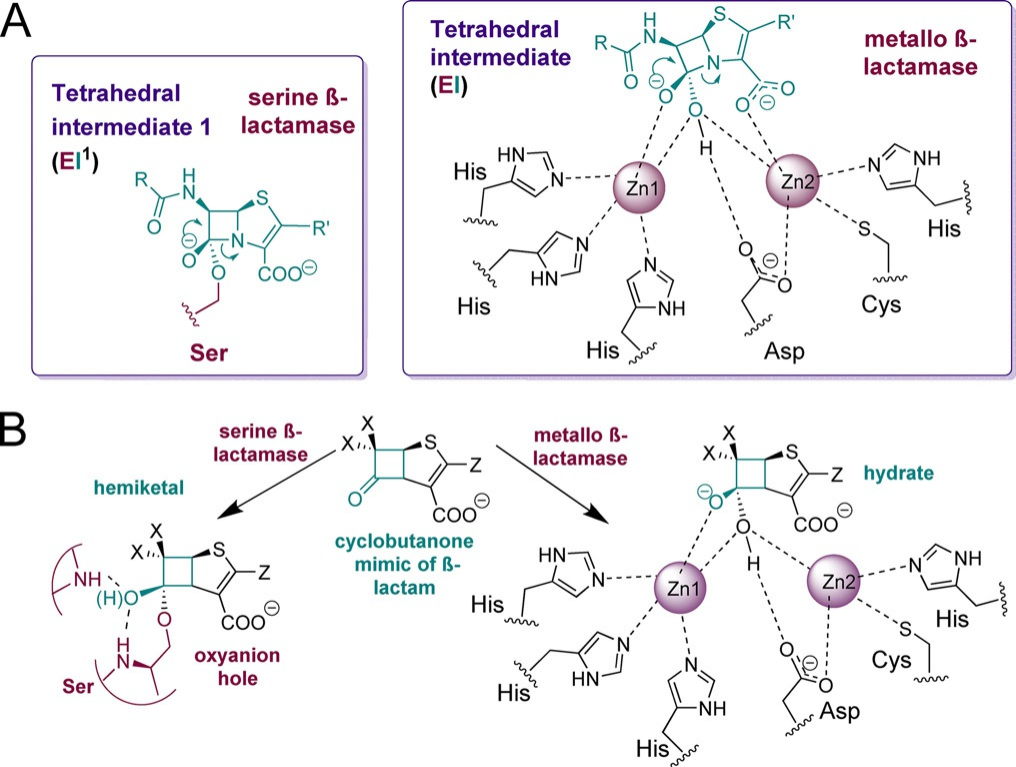
**A** Proposed binding modes of tetrahedral intermediates in the β‐lactamase‐catalyzed hydrolysis of a penem. **B** Cyclobutanones/penem analogues as potential broad‐spectrum SBL and MBL inhibitors.

The São Paulo MBL (SPM‐1) is widely distributed in South America, Europe and North America, in the Gram‐negative pathogen *Pseudomonas aeruginosa*.^9^ Like other B1 MBLs (NDM, VIM and IMP),^10^ SPM‐1 has a binuclear zinc center, but has loop characteristics of the B2 MBLs, suggesting it is a B1/B2 hybrid (Figures S2 and 3 in the Supporting Information), which, consequently, may be challenging to inhibit. To test the hypothesis that cyclobutanones can act as tetrahedral intermediate analogues for MBLs, we initiated studies on the binding mode of **1** to SPM‐1.

To study binding of **1** to SPM‐1, we initially employed ^19^F NMR (Figure S4 in the Supporting Information). SPM‐1 was selectively labeled at residue 152 on its α3 region, which forms part of the active site cleft, using cysteine alkylation by 3‐bromo‐1,1,1‐trifluoroacetone (BTFA) (Figure 3 A).^10^, ^11^, ^12^ The ^19^F NMR spectrum of labeled SPM‐1 (SPM‐1 Y152C*) manifests two peaks assigned as corresponding to “closed” (−83.3 ppm) and “open” (−72.4 ppm) conformations of the α3 loop (Figure S5).11a Addition of known MBL inhibitors (e.g., isoquinoline derivatives, 1,10‐*o*‐phenanthroline) results in line broadening and chemical shift changes in the ^19^F NMR of α3 variants.11a By contrast, titration of **1** with SPM‐1 Y152C* manifests only small effects on the SPM‐1 Y152C* ^19^F NMR spectra (Figure S5). We therefore employed a second BTFA‐labeled mutant, SPM‐1 Y58C*,11a incorporating a ^19^F label on the L3 loop that connects α3 and α4, and which is adjacent to the active site. The ^19^F NMR spectrum of SPM‐1 Y58C*11a has one major peak (−83.3 ppm; Figure 3 B). Addition of **1** (10 μm) causes a shift and line broadening, indicating **1** binds in the vicinity of Cys58 in a fast‐exchange manner relative to the NMR timescale. Monitoring the concentration dependence of ^19^F chemical shift changes on titration of **1** into SPM‐1 Y58C* enabled the *K*_D_ to be estimated as 22±7 μm.

**Figure 3:**
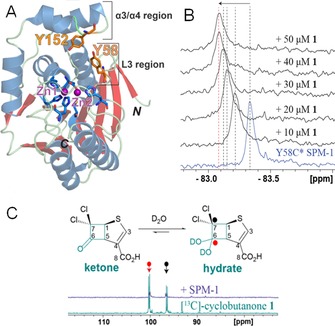
NMR reveals binding of cyclobutanone **1** to SPM‐1. A) View from an SPM‐1 crystal structure showing location of the ^19^F labels. B) ^19^F chemical shift changes for SPM‐1 Y58C* (45 μm) on titration with cyclobutanone **1**. C) NMR spectroscopy implies binding of **1** to SPM‐1 in its hydrated form. ^13^C NMR spectrum of **1** (4.2 mm) (green) and ^13^C NMR spectrum of **1** (4.2 mm) after addition of SPM‐1 (0.84 mm) (purple). Circles highlight peaks assigned to the hydrated form of **1**. Assays were buffered with 50 mm Tris‐D_11_, pH 7.5, in 9:1 H_2_O:D_2_O.

We then worked to obtain a structure of cyclobutanone **1** complexed to SPM‐1 by soaking crystals with excess inhibitor. SPM‐1 crystallized in its di‐zinc “closed” form,10a in which the α3 region folds over the active site, and in a previously unreported space group (P4_2_22). The resolution extended to 1.7 Å for uncomplexed crystals and 2.38 Å for inhibitor soaked crystals (Table S1 in the Supporting Information). Electron density maps for the latter indicated clear F_o_‐F_c_ difference density in the active site into which **1** was modelled as its hydrated form (Figure 4 A). SPM‐1, like other B1 MBLs, has a di‐zinc ion active site with one zinc ion bound at a (normally tetrahedral) tri‐histidine site (Zn1) and one in a trigonal bipyramidal site (Zn2) comprised of conserved Asp, Cys, and His residues (Figure S3). Wat1 “bridges” the zinc ions and is proposed to act as the “catalytic” nucleophile.10a Relative to uncomplexed SPM‐1 in the same crystal form (Table S1), a small (∼0.5 Å) movement of Zn2 into a more solvent‐exposed position in the active site is observed; however, the binding of **1** does not cause significant structural effects on the metal center with little change in Zn–Wat1; Zn1–Zn2 or Zn:protein ligand distances. Notable interactions made by **1** are in the vicinity of Zn2. The C4 carboxylate of **1** makes direct interactions with both Zn2 (2.48 Å distance) and Lys219 (2.91 Å; Figure 4 C), a binding mode likely involved in substrate carboxylate binding in most B1 MBLs (Figure S7 in the Supporting Information).^13^ By contrast, **1** makes only weak interactions with Zn1, with the two oxygen atoms of the C6 ketone hydrate with Zn1‐oxygen distances of 3.5 and 4.0 Å. In contrast to previous predictions about cyclobutanone binding to MBLs,^14^ the Zn‐bridging Wat1 is clearly present, potentially interacting with both the C6 oxygens of **1** (2.7 Å; Figure 4 C).

**Figure 4:**
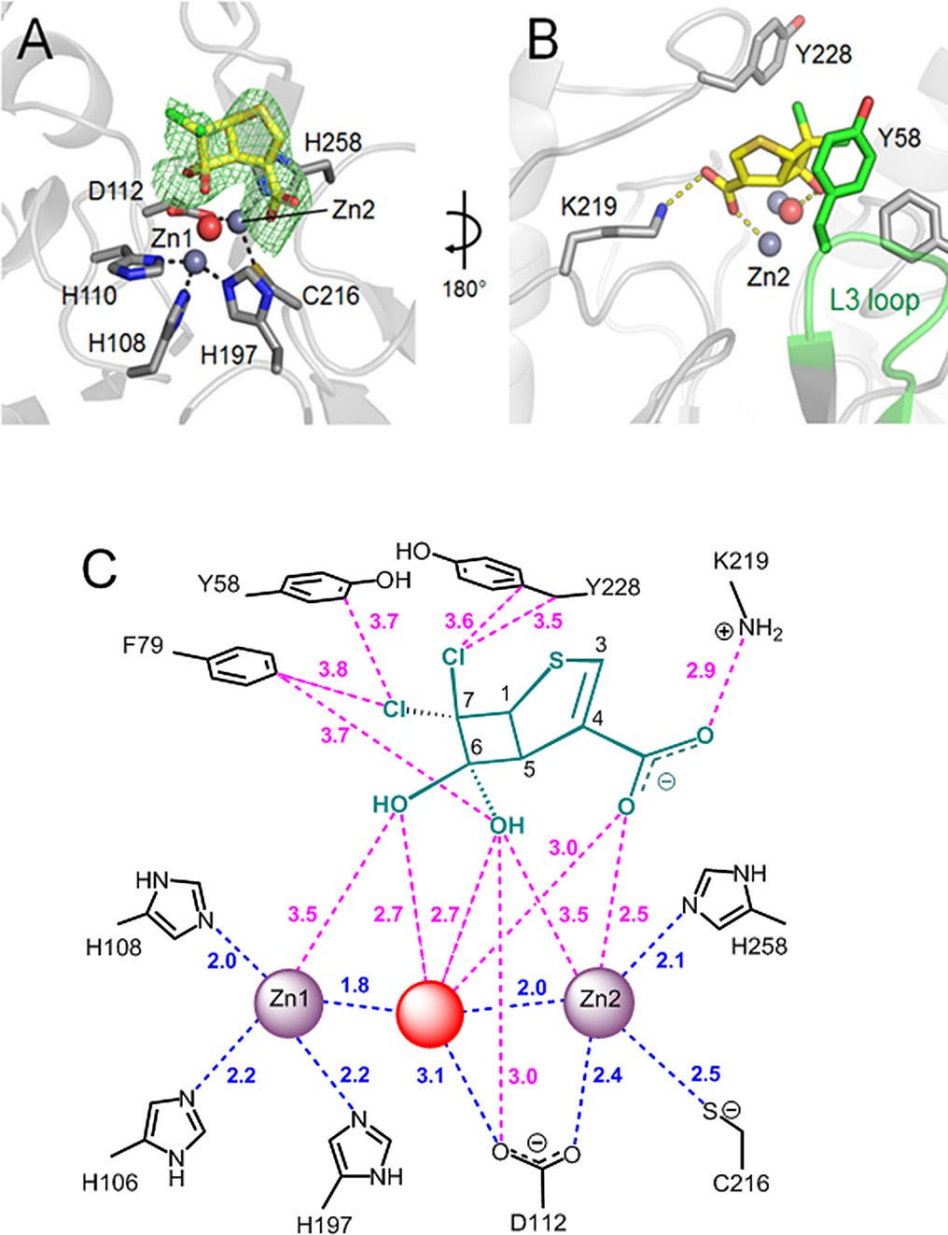
Binding mode of **1** to SPM‐1 as observed crystallographically. A**)** Cyclobutanone 1 (yellow) binding to the SPM‐1 active site of chain A (grey ribbon). F_o_‐F_c_ density (green, contoured at 3σ) calculated from the SPM‐1 model in the absence of ligand. Zinc ions and the bridging water/hydroxide (Wat1) are grey and red spheres, respectively. B) Interactions between SPM‐1 and cyclobutanone **1** (yellow dashes). C) Interactions of **1** with SPM‐1 in chain A. Distances between **1** (cyan) and the SPM‐1 active site are in magenta. Distances between active site atoms are in blue. The red sphere represents the bridging water molecule or hydroxide. Numbers indicate distances in Å.

SPM‐1 is distinguished from other B1 MBLs in possessing a “wall” of hydrophobic residues involving Phe57 and Tyr58 on its L3 loop, Phe79 on the loop connecting α5 and α1, and Phe151 and Tyr152 on the kinked α3 helix in the “closed” form, as well as Tyr228 on the opposite side of the active site cleft.^10^ Binding of **1** involves hydrophobic interactions with several of these, notably involving sandwiching of the bicyclic ring of **1** between the aromatic rings of Tyr58 (consistent with the ^19^F‐NMR results, Figure 3 B) and Tyr228, and edge‐face interactions of **1** with Phe79 (Figure 4 B). The Tyr152 side chain is more distant from **1**, with its OH group 6.5 (chain A) or 10.8 Å (chain B) away from the C7 atom of **1**, again consistent with the ^19^F NMR results (Figure S5). Notably, despite the clear presence of **1** at the active sites of both molecules in the asymmetric unit, the conformation of the α3 loop differs substantially in them (Figure S8 in the Supporting Information); compared to the rest of the structure, this region is flexible (B‐factors of 75 Å^2^, crystallographic chain A and 64 Å^2^, chain B) in support of the solution studies presented here and previously indicating α3 flexibility.10a, 11a By contrast, we do not see evidence for changes in the L3 loop on binding of **1**. The larger ^19^F NMR chemical shift changes observed on binding of **1** to SPM‐1 Y58C*, compared to SPM‐1 Y152C*, can be rationalized by the closer proximity of residue 58 to the inhibitor binding site, notwithstanding the more extensive conformational changes observed in the α3 region crystallographically.

Test refinements with the ketone and hydrated forms of **1** yielded similar statistics. We therefore used NMR to investigate the behavior of **1** in solution both in the presence and absence of SPM‐1. We synthesized **1** in which the C6 (ketone) and adjacent C7 dichlorinated carbon atoms were labeled with [^13^C]. Under the conditions of the binding studies, [^13^C]‐**1** was found to exist (almost) entirely as its hydrate (consistent with previous work),8a as indicated by the ^13^C chemical shift (102 ppm) for C6 and (98.7 ppm) C7, ^1^ *J_CC_*=38 Hz. Binding of **1** to SPM‐1 under these conditions was confirmed by ^13^C NMR, in which the peaks corresponding to hydrated **1** were reduced on addition of SPM‐1 (Figure 3 C). Addition of SPM‐1 to the sample yielded no change in the chemical shifts of either the C6 or C7 peaks, with no appearance of a peak around 190 ppm indicative of a ketone at C6. These results support the proposal that **1** is bound to SPM‐1 in its hydrated form, although we cannot exclude initial binding of the ketone form.

It is instructive to consider our results in the context of possible modes of β‐lactam binding, as our work represents the closest stable analogue of an intact β‐lactam for which an MBL complex structure is currently available. The structure implies substrate binding includes important interactions of the substrate carboxylate with Zn2 and the adjacent Lys219, as observed for hydrolyzed β‐lactams^13^ (Figure S7 in the Supporting Information) and β‐lactam analogues^15^ (Figure S9), as well as interactions with hydrophobic elements around the active site (Figure 4). The structure supports the involvement of these interactions in binding intact β‐lactam substrates, and, thus, in the formation of early‐stage complexes in catalysis.

It is notable that the complex with **1** does not feature strong interactions of the C6 oxygen atoms with Zn1. The Zn1 site is proposed to polarize the β‐lactam amide and participate in activation of the ‘hydrolytic“ water (Wat1).^16^ As such, it is proposed that binding of an intact β‐lactam triggers dissociation of Wat1 from Zn2 to generate a ”terminal“ hydroxide nucleophile on Zn1. The tetrahedral species formed by reaction of Wat1 with the β‐lactam carbonyl is stabilized by Zn1 binding, as supported by structural analysis of MBLs complexed with cyclic boronates, which are tetrahedral intermediate analogues (Figure S9 in the Supporting Information).^15^ In the hydrated cyclobutanone, the C6 oxygen atoms are likely protonated, possibly reducing affinity for Zn1 and instead favoring interaction with Wat1. Further, approach of the non‐polar C5−H bond of **1** to Zn2, which may be required to bring Zn1 and the C6 oxygen atoms into proximity, may be disfavored compared to that of β‐lactam derived amide nitrogen.

Inhibition of SBLs by cyclobutanones involves formation of a hemiketal linkage through reaction of the nucleophilic serine with the ketone and binding of the resultant tetrahedral species in the “oxyanion hole”.8a By contrast to the stabilization of tetrahedral intermediates by SBLs, MBLs are proposed to preferentially stabilize the ring‐opened anionic intermediates formed after addition of the water/hydroxide (Wat1) nucleophile to the β‐lactam carbonyl, but prior to proton transfer to the lactam‐derived nitrogen (Figure S1 in the Supporting Information). Interaction of Zn2 with the anionic nitrogen of such species is proposed to be important in intermediate stabilization.^16^ Our results, however, show that the SPM‐1 active site can bind with reasonable affinity to a species (i.e., a hydrated cyclobutanone) whose tetrahedral carbon (C6) atoms render it closely (though not perfectly) analogous to the oxyanion intermediate. Taking into consideration the activity of cyclobutanones and boronates against all classes of SBLs,8a, 17 these results indicate that structures mimicking the tetrahedral oxyanion merit investigation as starting points for potent inhibitors that are hydrolytically stable and effective against both SBLs and MBLs.

## Conflict of interest

The authors declare no conflict of interest.

## Supporting information

As a service to our authors and readers, this journal provides supporting information supplied by the authors. Such materials are peer reviewed and may be re‐organized for online delivery, but are not copy‐edited or typeset. Technical support issues arising from supporting information (other than missing files) should be addressed to the authors.

Supplementary
