# Supplementary material for: Cyclobutanone Mimics of Intermediates in Metallo‐β‐Lactamase Catalysis
Source: Chemistry. 2018 Jan 17;24(22):5734–7. doi: 10.1002/chem.201705886 (PMC5947706; doi:10.1002/chem.201705886)
Supplement: Supplementary file 1 — Supplementary [file CHEM-24-5734-s001.pdf]

CHEMISTRY
A European Journal

Supporting Information

Cyclobutanone Mimics of Intermediates in Metallo-β-Lactamase Catalysis

Martine I. Abboud,[a] Magda Kosmopoulou,[b] Anthony P. Krismanich,[c] Jarrod W. Johnson,[c] Philip Hinchliffe,[b] Jürgen Brem,[a] Timothy D. W. Claridge,[a] James Spencer,*[b] Christopher J. Schofield,*[a] and Gary I. Dmitrienko*[c]

chem_201705886_sm_miscellaneous_information.pdf

Cyclobutanone Mimics of Intermediates in Metallo-β-Lactamase Catalysis

Martine I. Abboud,[a] Magda Kosmopoulou,[b] Anthony P. Krismanich,[c] Jarrod W. Johnson,[c] Philip Hinchliffe,[b] Jürgen Brem,[a] Timothy D. W. Claridge,[a] James Spencer,[b] Christopher J. Schofield,[a] and Gary I. Dmitrienko[c]

[a]Department of Chemistry, University of Oxford, 12 Mansfield Road, Oxford OX1 3TA, United Kingdom

[b]School of Cellular and Molecular Medicine, University of Bristol, Medical Sciences Building, Bristol BS8 1TD, United Kingdom

[c]Department of Chemistry, University of Waterloo, 200 University Ave. W., Waterloo, Ontario, Canada N2L 3G1

Supporting Information

Contents

Materials and Methods **3**
Synthetic Experimental Procedures 3
General 3
Synthesis of cyclobutanone **1** 3
Method A 3
Method B 4
Synthesis of Doubly [^{13}C]-labelled cyclobutanone **1** 5
^{1}H and ^{13}C NMR Spectra 9
Protein Production and Purification 12
^{19}F Labeling of SPM-1 Variants and Method Validation 12
NMR Experiments 12
^{19}F NMR experiments 12
^{13}C NMR experiments 13
K_D fitting for cyclobutanone **1** binding to SPM-1 Y58C* 13
Protein Crystallization and Structure Determination 13
Supplementary Figures **15**
Figure S1. Outline mechanisms for metallo β-lactamase (MBL) and serine β-lactamase (SBL) catalysis showing proposed intermediates. 15
Figure S2. Views from MBL crystal structures highlighting potentially mobile regions. 16
Figure S3. View from an SPM-1 crystal structure. 17
Figure S4. Outline scheme of the labeling method and labeling validation steps.. 18
Figure S5. ^{19}F NMR spectra of Y152C* SPM-1 (lower trace, blue) and Y152C* SPM-**1** with increasing concentration of cyclobutanone **1** 19
Figure S6. Interactions of cyclobutanone **1** with SPM-1 active site as observed in chain B. 20
Figure S7. Comparative views from crystal structures of (A) hydrolyzed cefuroxime binding to NDM-1, a B1 MBL, (PDB ID: 4RL2)[13] and of (B) cyclobutanone **1** binding to SPM-1. 21
Figure S8. Conformations of SPM-1 in cyclobutanone-bound crystal structures. 22
Figure S9. Comparative overlaid views from crystal structures of a boronate inhibitor binding to the B1 MBL VIM-2 (PDB ID: 5FQC)[14] and of cyclobutanone **1** binding to SPM-1. 23
Supplementary Tables **24**
Table S1: Data Collection and Refinement Statistics 24
References **25**

Materials and Methods

Synthetic Experimental Procedures

General

Doubly-[^{13}C]-labelled sodium dichloroacetate (99 atom% ^{13}C) was from Cambridge Isotope Laboratories Inc. (Tewksbury, MA, USA). All other reagent chemicals were from Sigma-Aldrich Canada Co. (Oakville, ON, Canada) and were used without purification. Carbon tetrachloride was refluxed over P_2O_5 and distilled for immediate use. (+/-)-Ethyl 2,3-dihydrothiophene-3-carboxylate (**S3**) was prepared as described.[1] Reactions were monitored by thin-layer chromatography on aluminum-backed silica plates, with spots visualized by UV and basic $KMnO_4$ stain prepared according to a standard recipe. ^{1}H NMR spectra were recorded on a Bruker Avance 300 NMR spectrometer in $CDCl_3$, and acetone-d_6. Chemical shifts are reported in parts-per-million (ppm) relative to tetramethylsilane (TMS) and are calibrated to either TMS for spectra in $CDCl_3$, or to residual solvent proton peaks for acetone (2.05 ppm for residual proton; 206.6 ppm for solvent ^{13}C). Mass spectra were recorded by Dr. Richard Smith using either a Thermo Scientific Q-Exactive Orbitrap mass spectrometer for experiments requiring electrospray ionization or a JEOL HX110 Double Focusing mass spectrometer for electron impact ionization spectra in the University of Waterloo Mass Spectrometry Facility.

Synthesis of cyclobutanone 1:

The cyclobutanone **1** was prepared as a racemic mixture either via method A (as described previously)[1] or, more conveniently, via method B below.

(+/-)-7,7-Dichloro-2-thiabicyclo[3.2.0]hept-3-en-6-one-4-carboxylic acid (1).

***Method A*:**

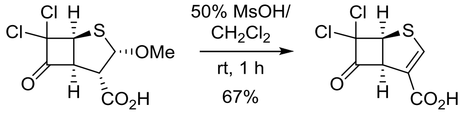

S2α/S2β 10:1 *dr* **1**

A mixture of the methoxy acids **2α** and **2β** (51.8 mg, 0.191 mmol, 10:1 *dr*) was stirred in a solution of 50% MsOH/CH_2Cl_2 (5 mL) at room temperature (rt) for 1 h. The solution was

diluted with EtOAc (50 mL) and washed with H_2O until the aqueous washes were no longer acidic (4 × 25 mL). The combined aqueous washes were back-extracted with EtOAc (2 × 25 mL) and CH_2Cl_2 (2 × 25 mL). The organic extracts were combined, dried over Na_2SO_4, and concentrated under reduced pressure to give the unsaturated acid **1** as an off-white solid (30.4 mg, 0.127 mmol, 67%). Mp 162–163 °C. ^{1}H NMR (300 MHz, acetone-d_6): δ 7.68 (d, $J_{3,5}$ = 1.8 Hz, 1H, H3), 5.62 (dd, $J_{5,1}$ = 10.0 Hz, $J_{5,3}$ = 1.8 Hz, 1H, H5), 5.23 (d, $J_{1,5}$ = 10.0 Hz, 1H, H1). ^{13}C NMR (75.5 MHz, acetone-d_6): δ 189.5, 162.3, 146.1, 123.3, 94.8, 72.8, 60.1. IR (film, cm^{-1}): br 3500–2300, 3066, 2925, 1807, 1700, br 1690–1630, 1558, 1446, 1338, 1258. LRMS (EI) *m/z* (relative intensity): 240 ([M(^{37}Cl^{35}Cl)]$^+$, 1.8), 238 ([M($^{35}Cl_2$)]$^+$, 2.1), 210 (20), 175 (100), 165 (30), 111 (20). HRMS (EI) *m/z*: 237.9258 calcd for $C_7H_4{}^{35}Cl_2O_3S$; 237.9252 obsd.

***Method B*:**

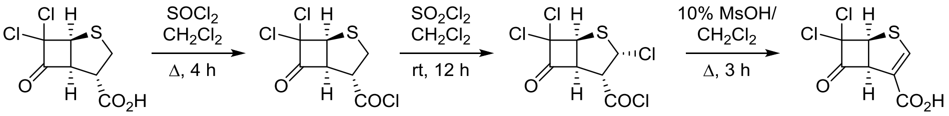

S3 **S4** **S5α** **1**
(94%, 3 steps)

Alternatively, **1** could be prepared in a one-pot three-step procedure from the acid **S3** via the acid chloride **S4**. $SOCl_2$ (4 mL, 55 mmol) was added to a solution of acid **S3** (1.475 g, 6.120 mmol) in CH_2Cl_2 (30 mL) and heated to reflux for 4 h. Concentration of the solution *in vacuo* provided the crude acid chloride **S4** as a yellow oil that was used directly in the next step without purification. ^{1}H NMR (300 MHz, $CDCl_3$): δ 5.03 (d, $J_{5,1}$ = 8.2 Hz, 1H, H5), 4.55 (d, $J_{1,5}$ = 8.2 Hz, 1H, H1), 4.03 (d, $J_{4,3\alpha}$ = 6.0 Hz, 1H, H4), 3.54 (d, J_{gem} = 13.1 Hz, 1H, H3α), 3.13 (dd, J_{gem} = 13.1 Hz, $J_{3\beta,4}$ = 6.0 Hz, 1H, H3β). ^{13}C NMR (75.5 MHz, $CDCl_3$): δ 192.9, 172.0, 89.1, 66.8, 60.3, 58.3, 35.4. IR (film, cm^{-1}): 2965, 1809, 1780, 1443, 997, 927, 873. LRMS (EI) *m/z* (relative intensity): 264 ([M($^{37}Cl_3$)]$^+$, 0.5), 262 ([M($^{37}Cl_2{}^{35}$Cl)]$^+$, 3), 260 ([M(^{37}Cl$^{35}Cl_2$)]$^+$, 7), 258 ([M($^{35}Cl_3$)]$^+$, 7), 225 (4), 223 (6), 200 (15), 198 (15), 141 (30), 131 (30), 85 (100), 55 (40). HRMS (EI) m/z: 257.9076 calcd for $C_7H_5{}^{35}Cl_3O_2S$; 257.9080 obsd. SO_2Cl_2 (550 μL, 6.85 mmol) was added dropwise over 2 min to a stirring solution of the crude acid chloride **S4** in CH_2Cl_2 (25 mL). After 12 h at rt, the reaction mixture was concentrated under reduced pressure to give the 3α-chloro acid chloride **S5α** as an off-white solid. ^{1}H NMR (300 MHz, $CDCl_3$): δ 6.03 (d, $J_{3,4}$ = 4.2 Hz, 1H, H3β), 5.13 (dd, $J_{5,1}$ = 8.1 Hz, $J_{5,4}$ = 5.2 Hz, 1H, H5), 4.99 (d, $J_{1,5}$ = 8.1 Hz, 1H, H1), 4.37 (dd, $J_{4,3}$ = 4.2 Hz, $J_{4,5}$ = 5.2 Hz, 1H, H4). ^{13}C NMR (75.5 MHz, $CDCl_3$): δ 190.5, 167.2, 84.7, 72.9, 69.4, 64.1, 60.0. MsOH (4

mL) was added to a solution of the crude acid chloride **S5α** in CH_2Cl_2 (36 mL) and the solution was heated to reflux for 3 h. The reaction mixture was cooled to rt, diluted with EtOAc (100 mL), then washed with H_2O (4 × 75 mL) until the aqueous washes showed a pH of 4. The combined aqueous washes were back-extracted with EtOAc (2 × 100 mL) and the combined organic extracts were dried over Na_2SO_4 and concentrated in vacuo to give the unsaturated acid **1** as a solid (1.368 g, 5.722 mmol, 94% over 3 steps) that was identical to the material prepared by method A as indicated by ^1H NMR analysis.

Synthesis of Doubly [^{13}C]-labelled cyclobutanone 1:

Racemic mixture of 6,7-di-[^{13}C]-labelled Ethyl (1*S*,4*S*,5*S*)- and (1*R*,4*R*,5*R*)-7,7-dichloro-6-oxo-2-thiabicyclo[3.2.0]heptane-4-carboxylate ([^{13}C]$_2$-S9)

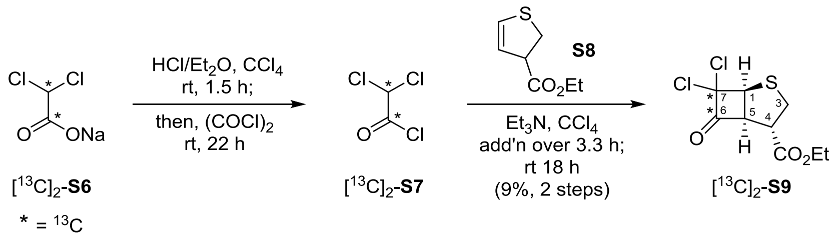

Doubly-[^{13}C]-labelled sodium dichloroacetate ([^{13}C]$_2$-**S6**) (407.7 mg, 2.67 mmol. 1 equiv) was suspended in dry freshly distilled carbon tetrachloride (8 mL) and stirred at rt under Ar. Hydrogen chloride in dry Et_2O (3.33 mL, 2 M, 2.67 mmol HCl, 1 equiv.) was added by syringe and the mixture was stirred at rt for 1.5 h. Oxalyl chloride (225.6 µL, 338.5 mg, 2.67 mmol, 1 equiv) was added by syringe and the mixture was stirred at rt overnight (22 h until start of use in next stage). Racemic ethyl 2,3-dihydrothiophene-3-carboxylate (**S8**) (91.4 mg, 0.578 mmol, 0.38 equiv), prepared as reported,[1] and Et_3N (743 µL, 91.4 mg, 5.33 mmol, 3.46 equiv) were dissolved and stirred in dry CCl_4 (7 mL) in a 25 mL round-bottom flask under Ar. The reaction mixture was drawn into a 10 mL polypropylene syringe. A syringe filter (Pall GMP acrodisk, 0.45 µm, 2.5 cm dia.) and a stainless steel pipetting needle were attached, and the contents were filtered into an empty 10 mL polypropylene syringe, which was mounted in an inverted position, under gentle flow of Ar through a three-way plastic Luer syringe valve. A 6-inch stainless steel needle was attached to the freshly charged syringe, which was then mounted in an automated syringe pump, with the needle piercing the septum of the flask. The headspace gas was expelled from the syringe, and the addition was started at a rate of 2.5 mL/h (3 h 18 m calculated addition time). Once addition began, the needle was submerged in the reaction medium to prevent the formation of polymeric products

at the needle tip. After addition, the reaction was stirred overnight (21 h). The reaction was diluted with EtOAc and filtered through Celite®. The filtrate was concentrated and dissolved in EtOAc (80 mL) and the solution was washed with 2 M aq. HCl (25 mL), water (50 mL; brine (10 mL) added to disrupt emulsion), dried (Na_2SO_4), and concentrated. Chromatography on silica (230–400 mesh) with 5:95 Et_2O:hexanes gave 14.4 mg (9.2%) of the title compound [^{13}C]$_2$-**S9** as a thick oil. ^{1}H NMR (300 MHz, $CDCl_3$): δ 5.06 (1 H, m), 4.52 (1H, td, *J* = 8.3, 2.3 Hz), 4.21 (2 H, qd, *J* = 7.2, 0.7 Hz), 3.65 (1 H, br t, *J* = 5.4 Hz), 3.45 (1 H, dt, *J* = 12.4, 1.2 Hz), 3.05 (1 H, dd, *J* = 12.4, 5.8 Hz), 1.29 (3 H, t, *J* = 7.2 Hz); ^{13}C NMR (75.5 MHz, $CDCl_3$) δ 194.87 (d, *J* = 34.0 Hz, ^{13}C-6 **C**O), 169.8, 89.26 (d, *J* = 34.0 Hz, ^{13}C-7 **C**Cl_2), 67.5, 62.1, 58.9, 50.1, 35.6, 14.1.

Racemic mixture of 6,7-di-[^{13}C]-labelled (1*S*,4*S*,5*S*)- and (1*R*,4*R*,5*R*)-7,7-dichloro-6-oxo-2-thiabicyclo[3.2.0]heptane-4-carboxylic acid ([^{13}C]$_2$-S3)

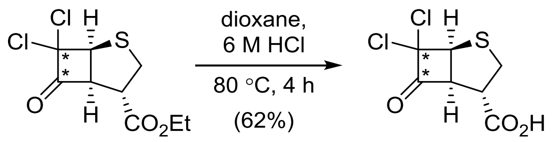

[^{13}C]$_2$-**S9** [^{13}C]$_2$-**S3**

To a solution of the ester [^{13}C]$_2$-**S4** (41.4 mg, 0.053 mmol) in dioxane (2 mL) was added 4 mL of 6 M aq. HCl, and the reaction was stirred at 80 °C for 4 h. The reaction mixture was transferred to a separating funnel; water (4 mL) and brine (5 mL) were then added. The mixture was washed with CH_2Cl_2 (5 × 15 mL), and the combined organic portions were dried (Na_2SO_4) and concentrated. The crude product, a fine beige solid, contained a small amount of apparent phthalate ester impurity (by ^{1}H NMR). The material was suspended in hexane (3 mL) and briefly exposed to an ultrasonic bath. After the solids had settled, the hexane was carefully withdrawn and the process was repeated. The remaining solid was dried to give 8.0 mg (62%) of [^{13}C]$_2$-**S3**, which was used directly in the following step. ^{1}H NMR (300 MHz, acetone-d_6): δ 11.07–10.8 (1 H, br), 5.22–5.14 (1 H, m), 4.69 (1 H, tdd, *J* = 8.3, 2.0, 0.6 Hz), 3.81 (1 H, m), 3.49 (1 H, dt, *J* = 12.4, 1.1 Hz), 3.05 (1 H, dd, *J* = 12.4, 5.6 Hz); ^{13}C NMR (75.5 MHz, acetone-d_6): δ 196.13 (d, *J* = 33.1 Hz, ^{13}C-6 **C**O), 90.28 (d, *J* = 33.1 Hz, ^{13}C-7 **C**Cl_2).

Racemic mixture of 6,7-di-[^{13}C]-labelled (1*S*,5*S*)- and (1*R*,5*R*)-7,7-dichloro-6-oxo-2-thiabicyclo[3.2.0]hept-3-ene-4-carboxylic acid ([^{13}C]$_2$-1)

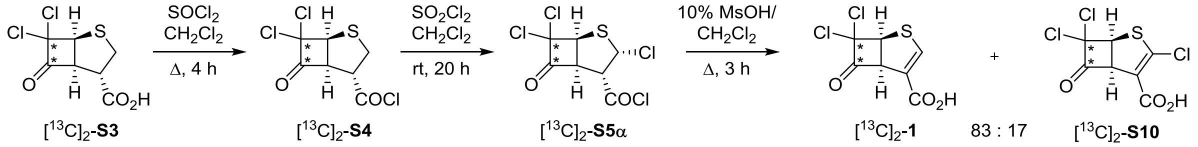

The carboxylic acid [^{13}C]$_2$-**S3** (8.0 mg, 0.033 mmol) was dissolved in CH_2Cl_2 (2 mL) in a 25 mL round-bottom flask and $SOCl_2$ (1 mL, 13.7 mmol, 416 equiv.) was added. The mixture was stirred and maintained at 70 °C for 4 h. The mixture was concentrated in vacuo, dissolved in CH_2Cl_2 (2 mL), SO_2Cl_2 (0.2 mL, 2.47 mmol, 75 equiv.) was added and the reaction was purged with Ar and sealed for overnight stirring at rt. After 20 h, the reaction was concentrated (rotary evaporation at $<$40 °C, and brief exposure to high vacuum at rt), and dissolved in CH_2Cl_2 (2 mL); MsOH (3 drops) was then added. The reaction was stirred at reflux (60 °C bath temp.) for 3 h, cooled to rt, diluted with EtOAc and washed with H_2O (7 × 1 mL). The organic phase was dried (Na_2SO_4) and concentrated to give a mixture (7.8 mg) of the intended product [^{13}C]$_2$-**1** (83 mol % by ^{1}H NMR integration) contaminated with a chlorinated by-product impurity [^{13}C]$_2$-**S10** (17 mol %), which was used in β-lactamase studies without purification. [^{13}C]$_2$-**1**: ^{1}H NMR (300 MHz, acetone-*d*$_6$): δ 7.68 (1 H, m), 5.62 (1 H, dddd, J = 12.3, 10.1, 5.4, 1.9 Hz), 5.23 (1 H, dddd, J = 10.1, 6.4, 1.8, 0.4 Hz); ^{13}C NMR (75.5 MHz, acetone-*d*$_6$): δ 188.6 (d, J = 30.1 Hz, ^{13}C-6 **C**O), 93.9 (d, J = 30.1 Hz, ^{13}C-7 **C**Cl$_2$); HRMS (−ESI) *m/z*: 238.92525 calcd for $C_5{}^{13}C_2H_3O_3{}^{35}Cl_2S$; 238.92672 obsd. Byproduct [^{13}C]$_2$-**S10**: ^{1}H NMR (300 MHz, acetone-*d*$_6$): δ 5.74 (1 H, ddd, J = 11.3, 10.4, 5.3 Hz), ~5.2 (1 H; signal partially overlapped with the C1-H signal from the main component [^{13}C]$_2$-**1**).

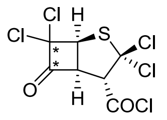

[^{13}C]$_2$-**S11**
not observed

likely precursor to [^{13}C]$_2$-**S10**

This impurity was not observed in the synthesis of unlabeled **1** and likely arises from the use of larger quantities (in relation to substrate) of thionyl chloride and sulfuryl chloride in the preparation of labelled **1** compared to larger preparations of the unlabeled compound. The larger reagent excesses and longer reaction time likely enabled production of a small

quantity of tetrachlorinated intermediate [$^{13}C]_2$-**S11**, giving rise to undesired chloroalkene product [$^{13}C]_2$-**S10**, which is not seen in larger-scale preparations of unlabeled material.

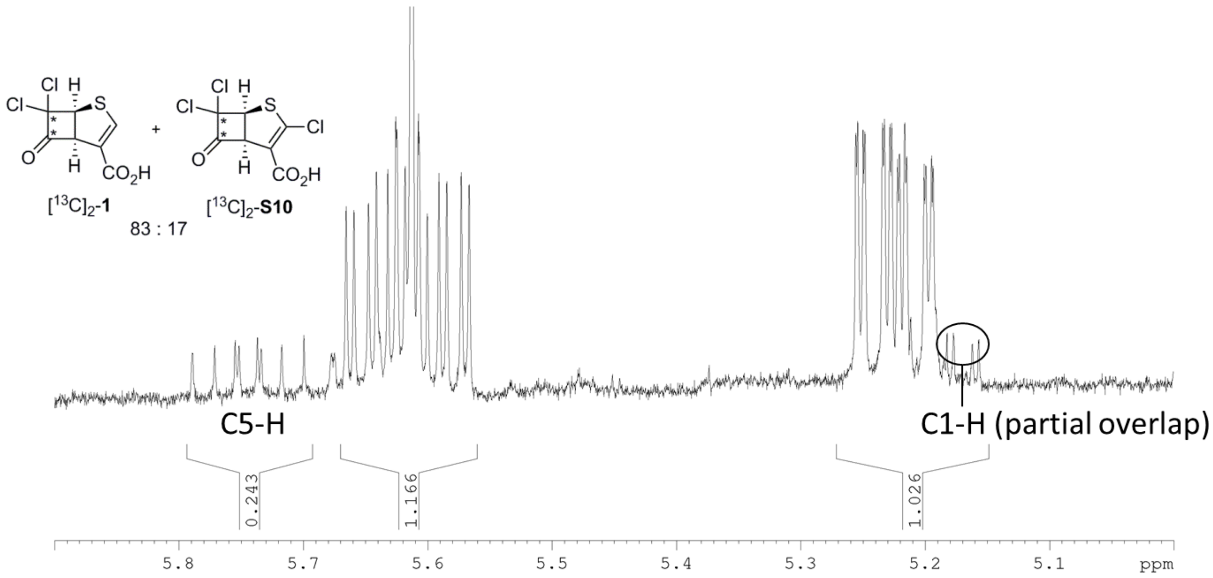

Expanded ^{1}H NMR spectrum of the doubly labelled cyclobutanone [$^{13}C]_2$-**1**, showing its complex signals due to H–^{13}C coupling. The over-chlorinated by-product of the reaction [$^{13}C]_2$-**S10** integrates to 17 mol%.

The bridgehead protons, H1 and H5, for [$^{13}C]_2$-**1** and [$^{13}C]_2$-**S10** are shown in the expansion of the ^{1}H NMR spectrum above (Figure S1). Because of the absence of the C3 proton in [$^{13}C]_2$-**S10**, the two proton signals for H1 and H5 exhibit simplified splitting patterns (ddd) in comparison to their counterpart protons in [$^{13}C]_2$-**1** (dddd), while still possessing the same vicinal proton-proton and two- and three-bond couplings to the [^{13}C]-enriched carbon atoms.

^{1}H and ^{13}C NMR Spectra

6,7-Di-[^{13}C]-labelled Ethyl (1*S*,4*S*,5*S*)- and (1*R*,4*R*,5*R*)-7,7-dichloro-6-oxo-2-thiabicyclo[3.2.0]heptane-4-carboxylate ([^{13}C]$_2$-S9)

($CDCl_3$; ^{1}H: 300 MHz; ^{13}C: 75 MHz)

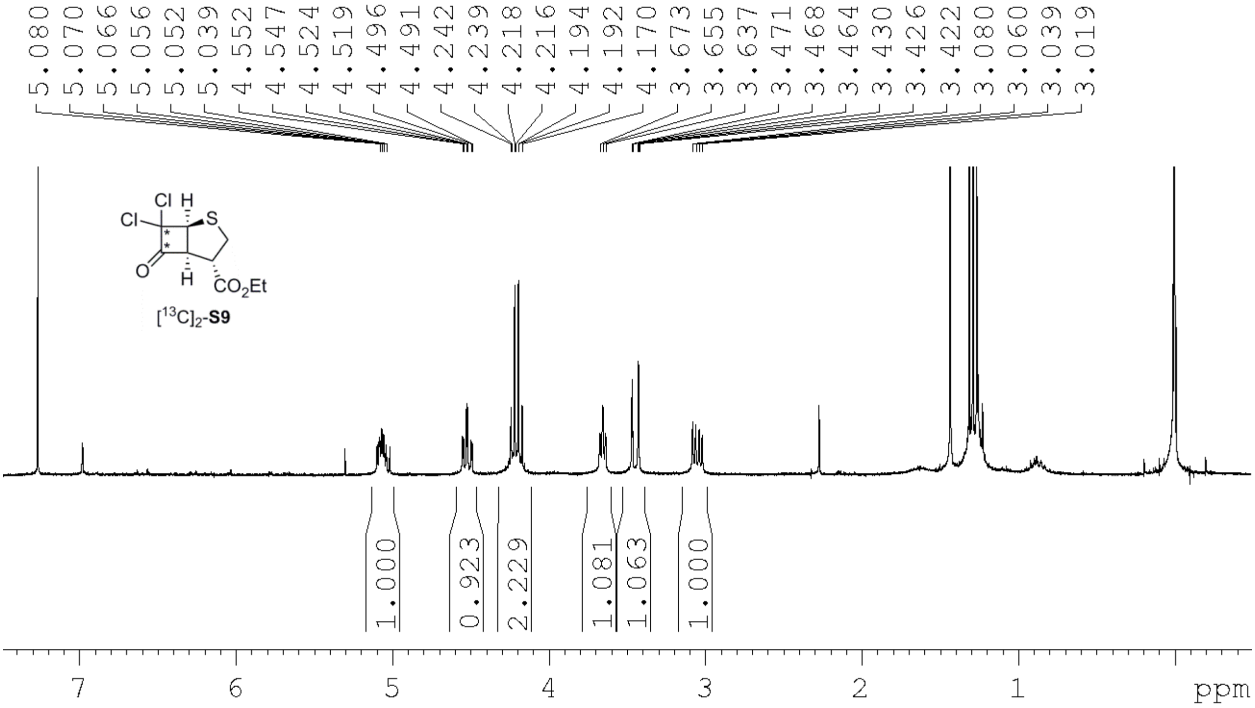

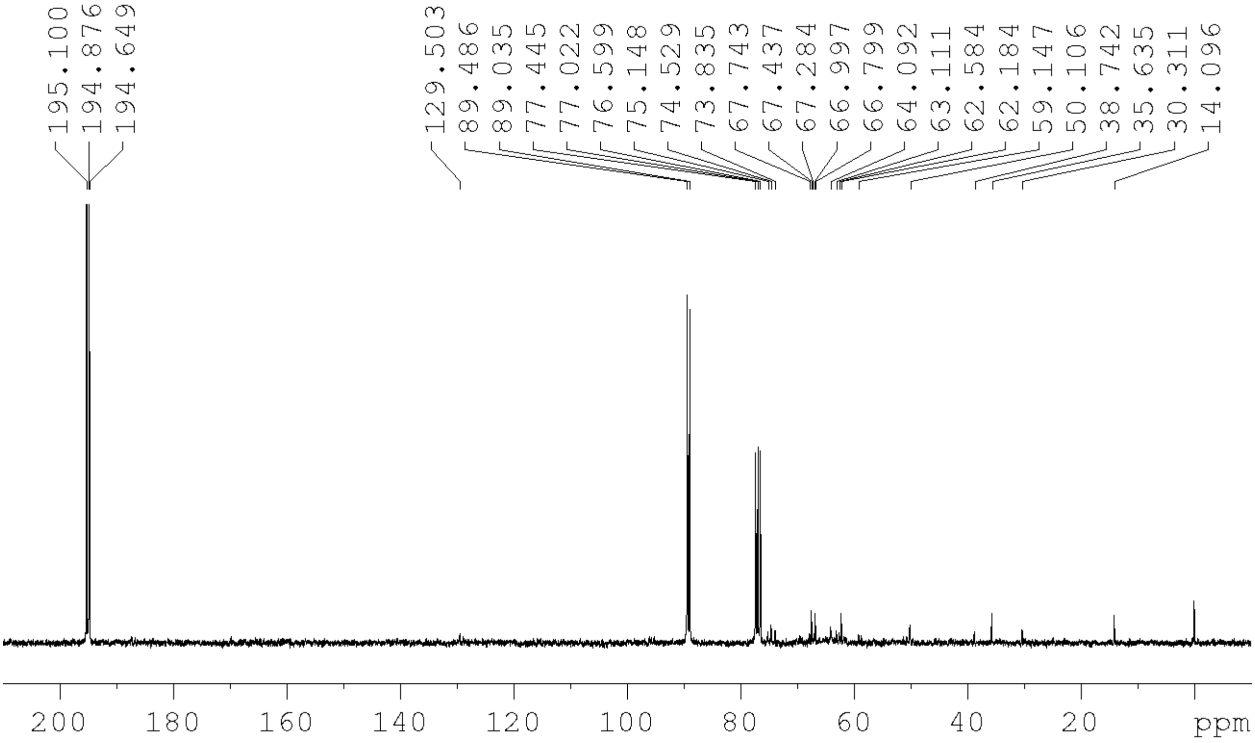

6,7-Di-[^{13}C]-labelled Ethyl (1*S*,4*S*,5*S*)- and (1*R*,4*R*,5*R*)-7,7-dichloro-6-oxo-2-thiabicyclo[3.2.0]heptane-4-carboxylate ([^{13}C]$_2$-S9)

(acetone-d_6; ^{1}H: 300 MHz; ^{13}C: 75 MHz)

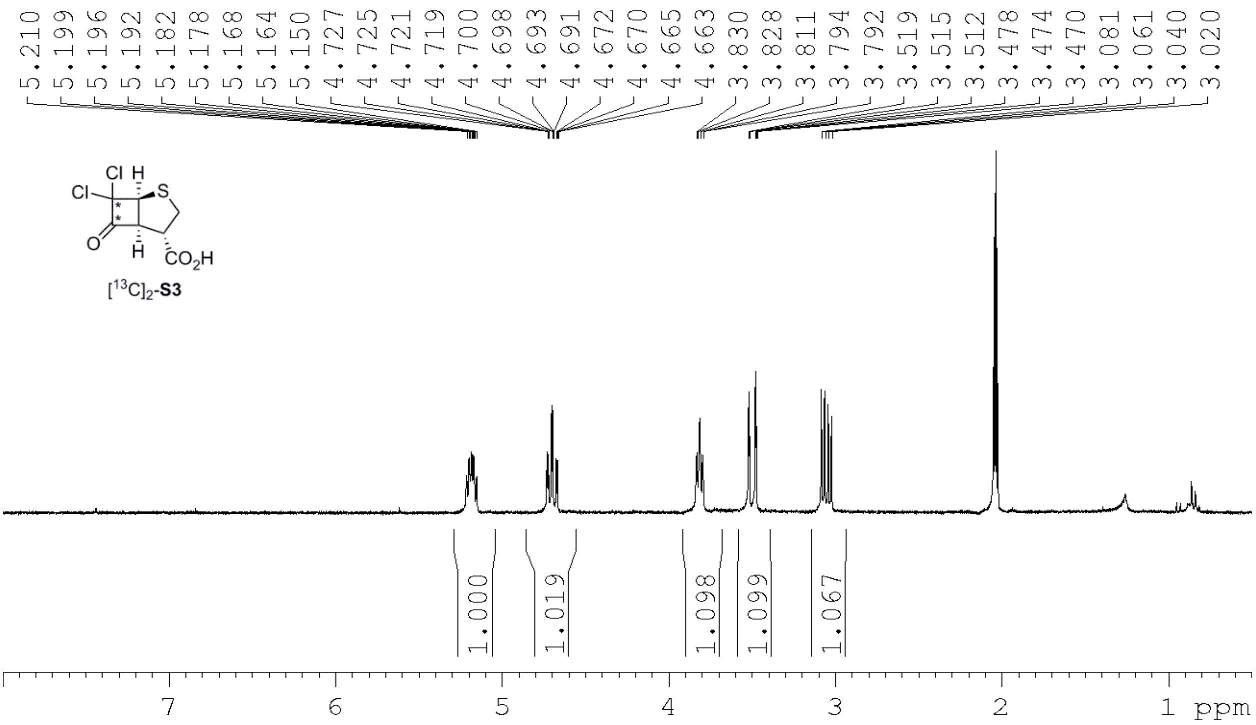

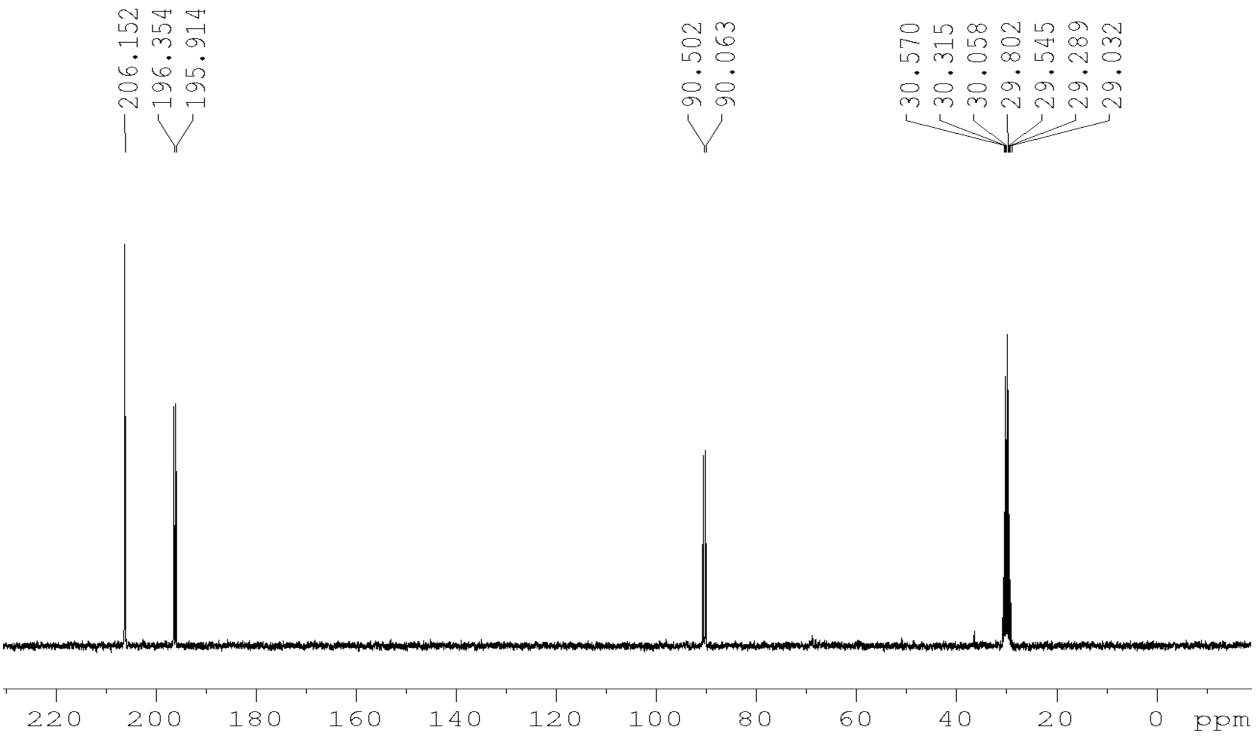

6,7-Di-[^{13}C]-labelled (1*S*,5*S*)- and (1*R*,5*R*)-7,7-dichloro-6-oxo-2-thiabicyclo[3.2.0]hept-3-ene-4-carboxylic acid ([^{13}C]$_2$-1)

(acetone-d_6; ^{1}H: 300 MHz; ^{13}C: 75 MHz)

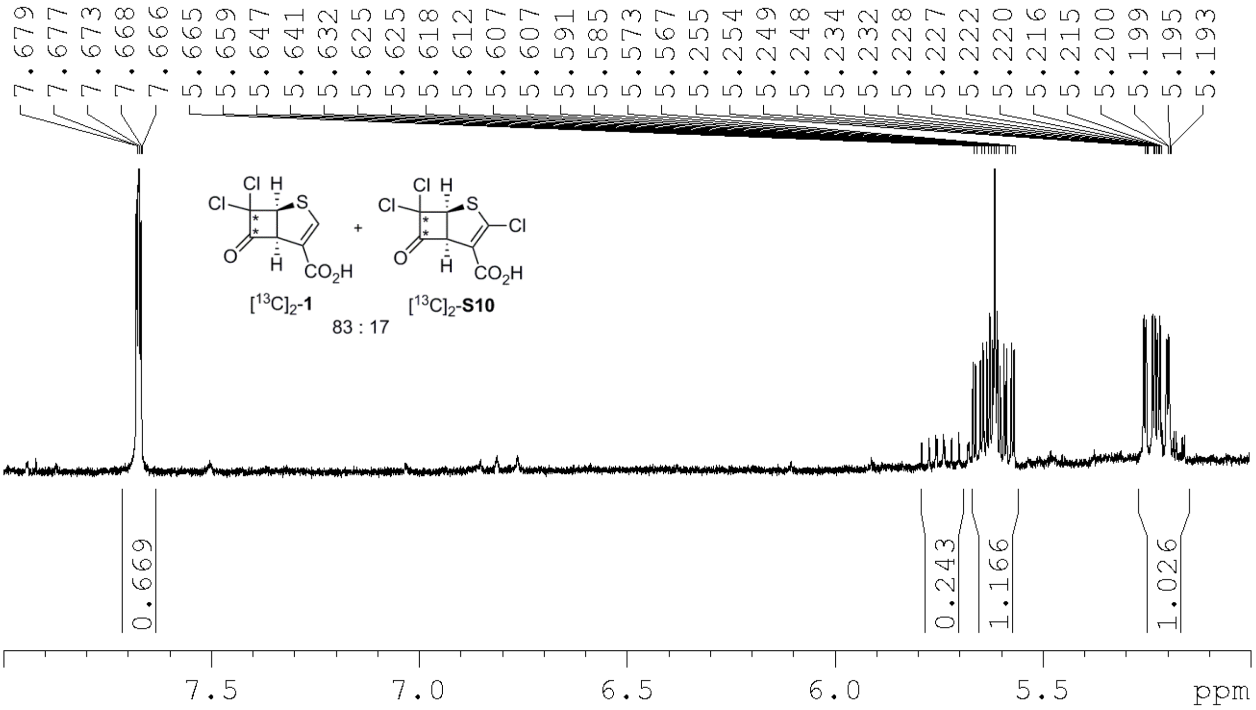

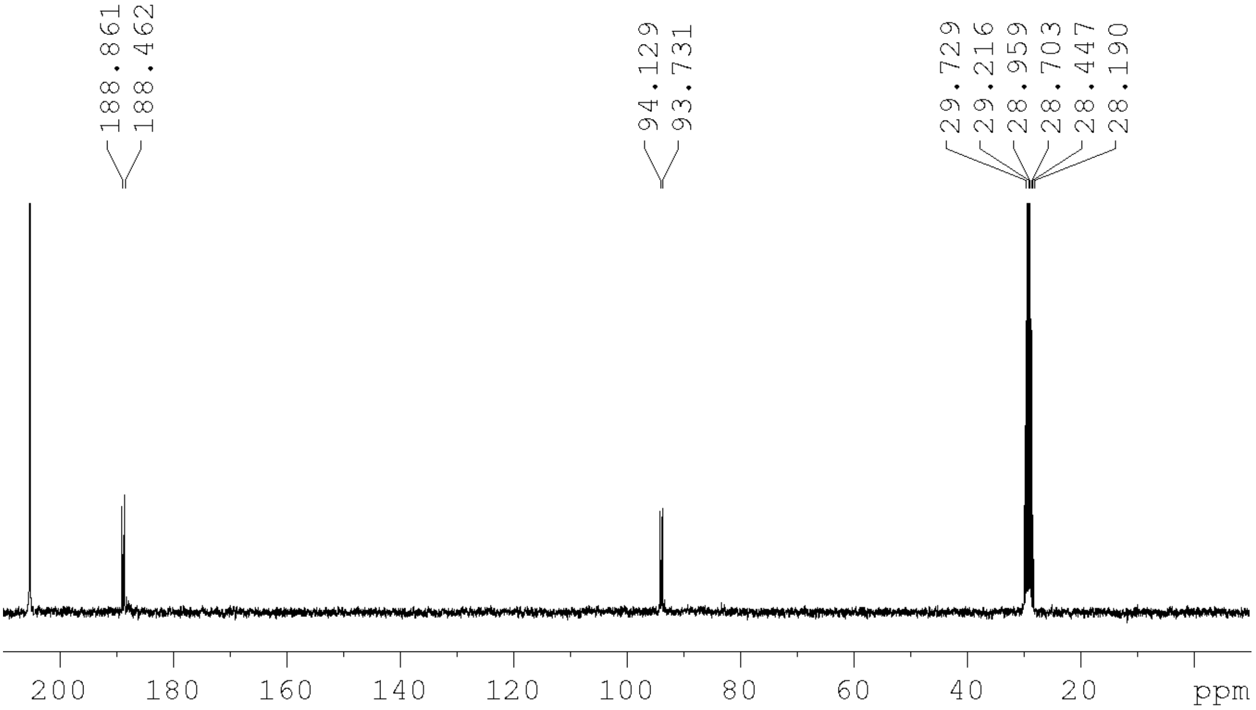

Protein Production and Purification

Recombinant wildtype (wt) SPM-1, Y58C SPM-1 and Y152C SPM-1 proteins were expressed and purified as previously described.[2] In summary, SPM-1 and variants were expressed in *E. coli* BL21 (DE3) pLysS grown in 2YT media supplemented with 50 μg/mL ampicillin and 50 μg/mL chloramphenicol. Cells were grown at 37 ºC until OD_{600} reached ~ 0.6-0.8. After induction with isopropyl-β-D-thiogalactopyranoside (IPTG, 0.5 mM), the temperature was reduced to 30 ºC for 6 h. Cells were harvested and recombinant SPM-1 proteins purified following a 3-step purification protocol as reported.[2]

^{19}F Labeling of SPM-1 Variants and Method Validation

Purified SPM-1 Y58C and SPM-1 Y152C were treated with tris-(2-carboxyethyl)phosphine (TCEP, 2 mM), then incubated on ice (5 min) and buffer exchanged (PD-10 Sephadex G-25 desalting column (GE Healthcare)) into phosphate buffer (50 mM, pH 7.0, 200 mM NaCl). A concentrated stock of 3-bromo-1,1,1-trifluoroacetone (100 mM) in phosphate buffer was freshly prepared prior to the reaction. Samples of SPM-1 variants (0.15 mM) were treated with 3-bromo-1,1,1-trifluoroacetone (2 mM) and incubated for 10 min at room temperature, prior to buffer exchange into Tris buffer (50 mM, pH 7.5, 200 mM NaCl). The selective incorporation of the label was confirmed by trypsin digestion and MALDI-ToF MS analyses. Circular dichroism and activity assays were followed to confirm that labeling did not affect protein's secondary structure content and activity, respectively. The method validation steps are summarized in **Figure S4** as previously reported.[2a]

NMR Experiments

^{19}F NMR experiments

^{19}F NMR spectra were recorded using a Bruker AVIII 600 MHz NMR spectrometer equipped with a BB-^{19}F/^{1}H Prodigy N_2 cryoprobe using 5 mm diameter NMR tubes (Norell). Samples contained the ^{19}F-labeled di-Zn(II)-SPM-1 complex (45 μM) in Tris buffer (50 mM, pH 7.5) supplemented with 10% D_2O and 200 mM NaCl, unless otherwise stated. Cyclobutanone analogue was titrated in the assay mixture from a DMSO stock. Spectra were

typically obtained using 512 scans. Trifluoroacetic acid (50 μM) was used as an internal NMR standard. Data were processed using TopSpin 3.1 software (Bruker).

^{13}C NMR experiments

NMR experiments were carried out using a Bruker AVIII 700 spectrometer equipped with an inverse TCI cryoprobe optimized for ^{1}H observation and installed with Topspin 3.1 software (Bruker). The assay mixture contained wt SPM-1 (0.84 mM) and [^{13}C]-labeled cyclobutanone analogue **1** (4.2 mM) in Tris-D_{11} buffer (50 mM, pH 7.5) supplemented with 0.02 % NaN_3 and 10% D_2O.

K_D fitting for cyclobutanone 1 binding to SPM-1 Y58C*

The K_D value for binding to SPM-1 Y58* of cyclobutanone **1** was determined by fitting ^{19}F chemical shift using the equation below, where $[L_0]$ is the total ligand (**1**) concentration and $[P_0]$ is the total protein (SPM-1 Y58C*) concentration. *y* was plotted as the fractional chemical shift change (observed chemical shift Δobs / maximal chemical shift Δmax).[2a]

$$y = \frac{([L_0] + [P_0] + K_D) - \sqrt{([L_0] + [P_0] + K_D)^2 - 4[L_0][P_0]}}{2[P_0]}$$

Protein Crystallization and Structure Determination

SPM-1 was crystallized by sitting drop vapor diffusion from 1.0 M lithium sulfate, 1.6 M ammonium sulfate. Crystallization experiments were set up using a Phoenix crystallization robot (Art-Robbins, Sunnyvale, CA, U.S.A.). Crystals were cryoprotected by transient exposure to reservoir solution plus 25 % glycerol before freezing by plunging into liquid nitrogen and storage at 100 K. For the cyclobutanone complex crystals were soaked in ligand (5 mM) for a period of 30 minutes before freezing. Diffraction data were collected at beamline I03 of Diamond Light Source (Didcot, U.K.). X-ray data sets were indexed and integrated using XDS[3] or Mosflm,[4] and scaled using Aimless in the CCP4 suite.[5] Structures were solved by molecular replacement using Phaser,[6] using the native SPM-1 structure (PDB ID: 4BP0)[2b] as a search model, and completed by iterative rounds of manual model building in Coot[7] and refinement in Phoenix.[8] Structure validation was assisted by

Molprobity[9] and Phenix.[8] Figures were prepared using PyMOL (www.pymol.org). Co-ordinates and structure factors have been deposited with the Protein Data Bank (www.rcsb.org/pdb) with accession numbers 5NDE and 5NDB for the unliganded and cyclobutanone-bound structures, respectively.

Supplementary Figures

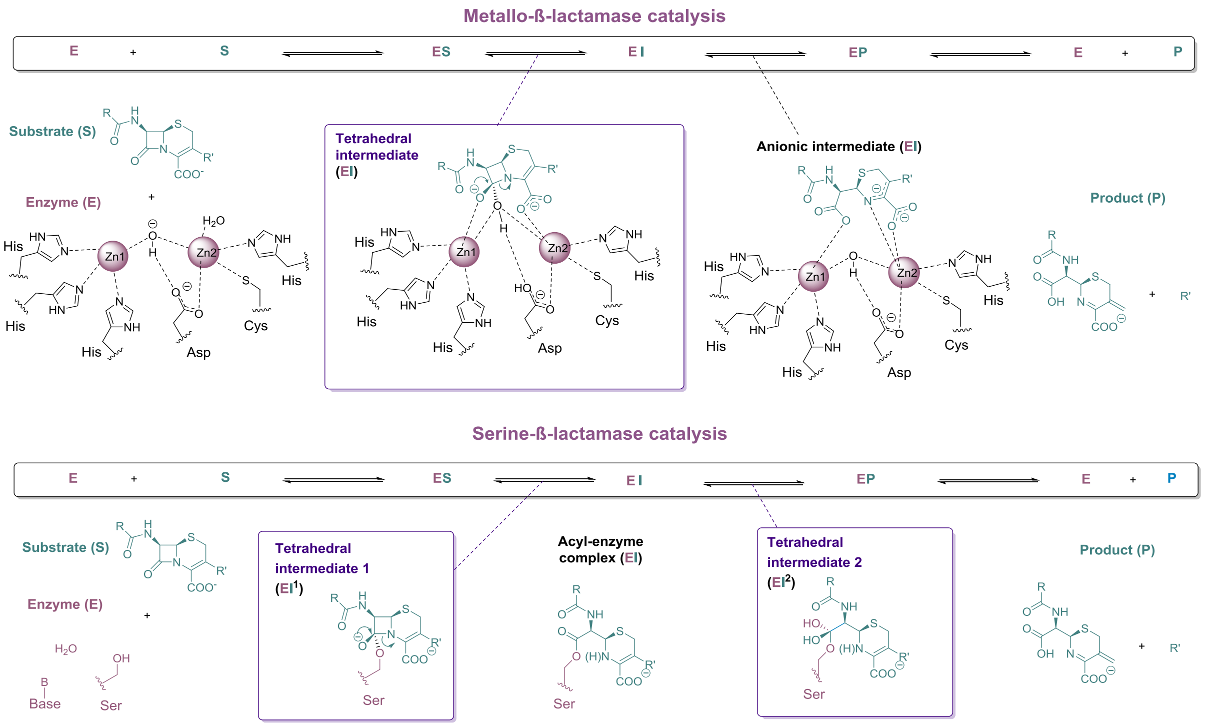

Figure S1. Outline mechanisms for metallo β-lactamase (MBL) and serine β-lactamase (SBL) catalysis showing proposed intermediates.[10]

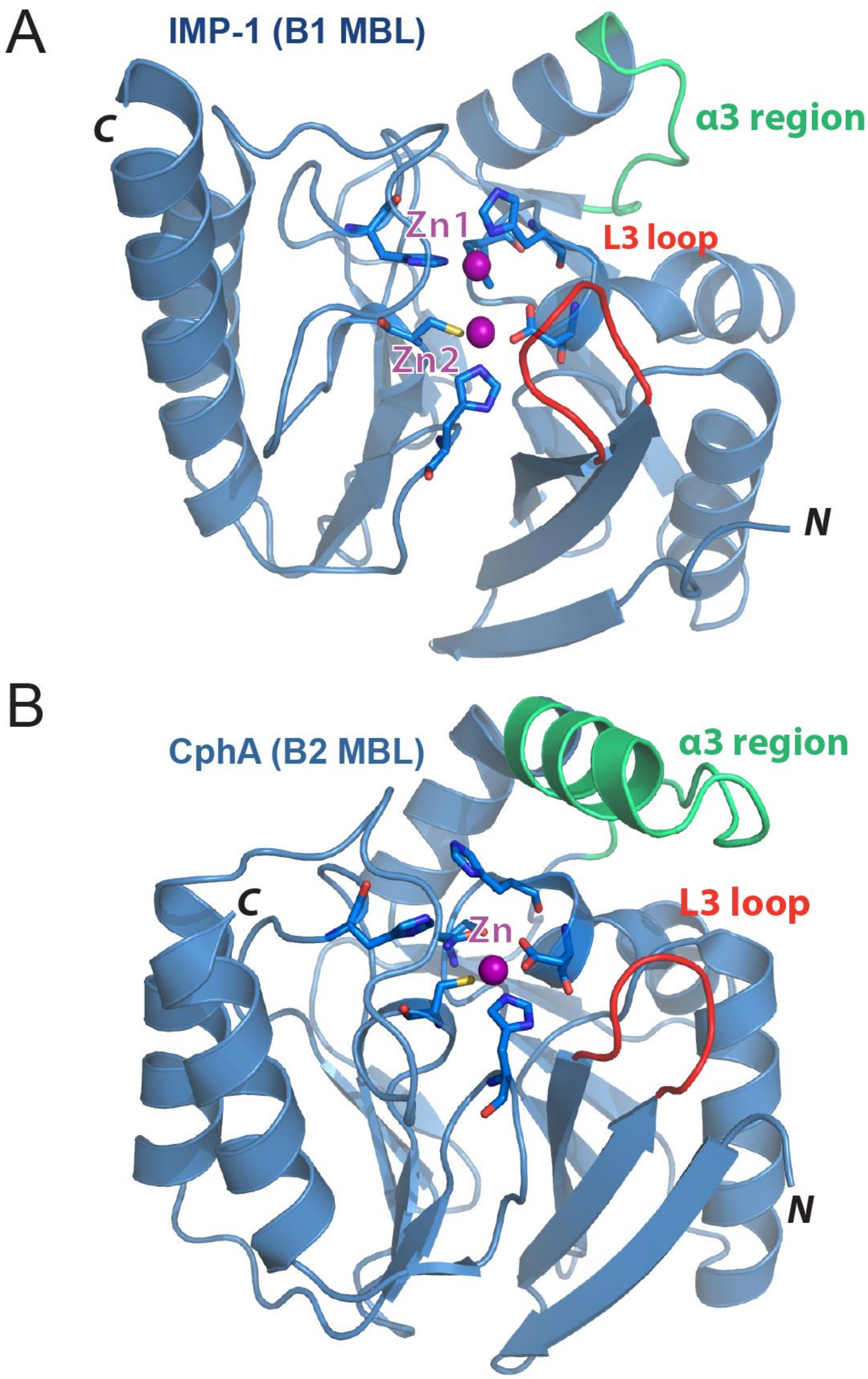

Figure S2. Views from MBL crystal structures highlighting potentially mobile regions. Views of **(A)** IMP-1 (di-zinc ion B1 MBL, PDB ID: 1JJT)[11] and **(B)** CphA (mono-zinc ion B2 MBL, PDB ID: 1X8I)_ENREF_4[12] highlighting the different mobile regions (L3 loop (red), and α3 region (green)) that characterize the MBL subfamilies. A longer L3 loop (red) is characteristic of the di-Zn B1 MBLs. The B2 MBLs using mono-zinc ion are characterized by an elongated α3 region (green) and a shorter L3 loop (red).

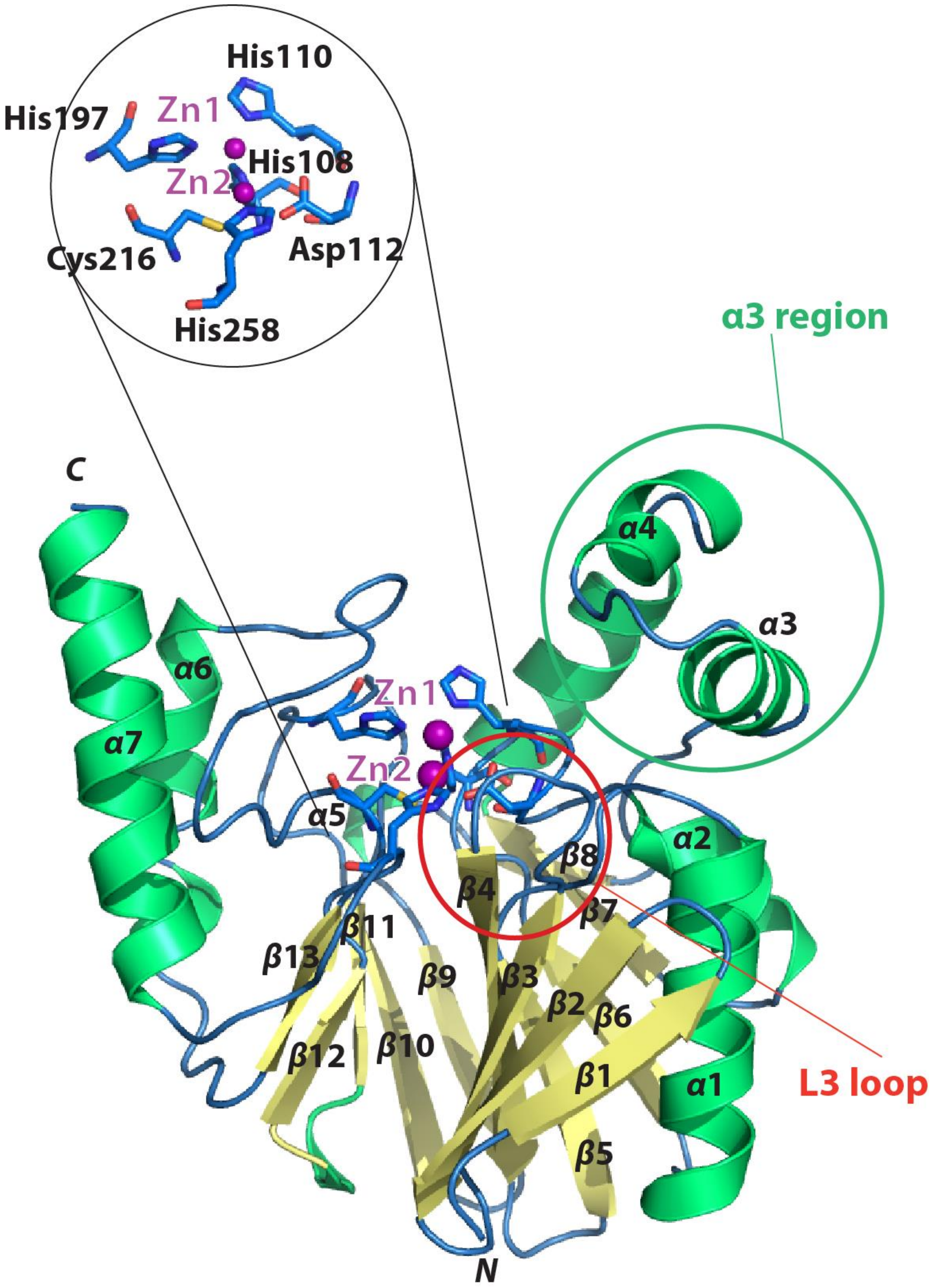

Figure S3. View from an SPM-1 crystal structure. The labeled regions, α3 and L3, are highlighted in green and red, respectively. Selected active site residues are shown as sticks in the highlighted red circle. The first Zn(II) is coordinated by 3 His residues: H108, H110 and H197. The second Zn(II) is coordinated by D112, H258 and C216. The figure was created using PyMOL.

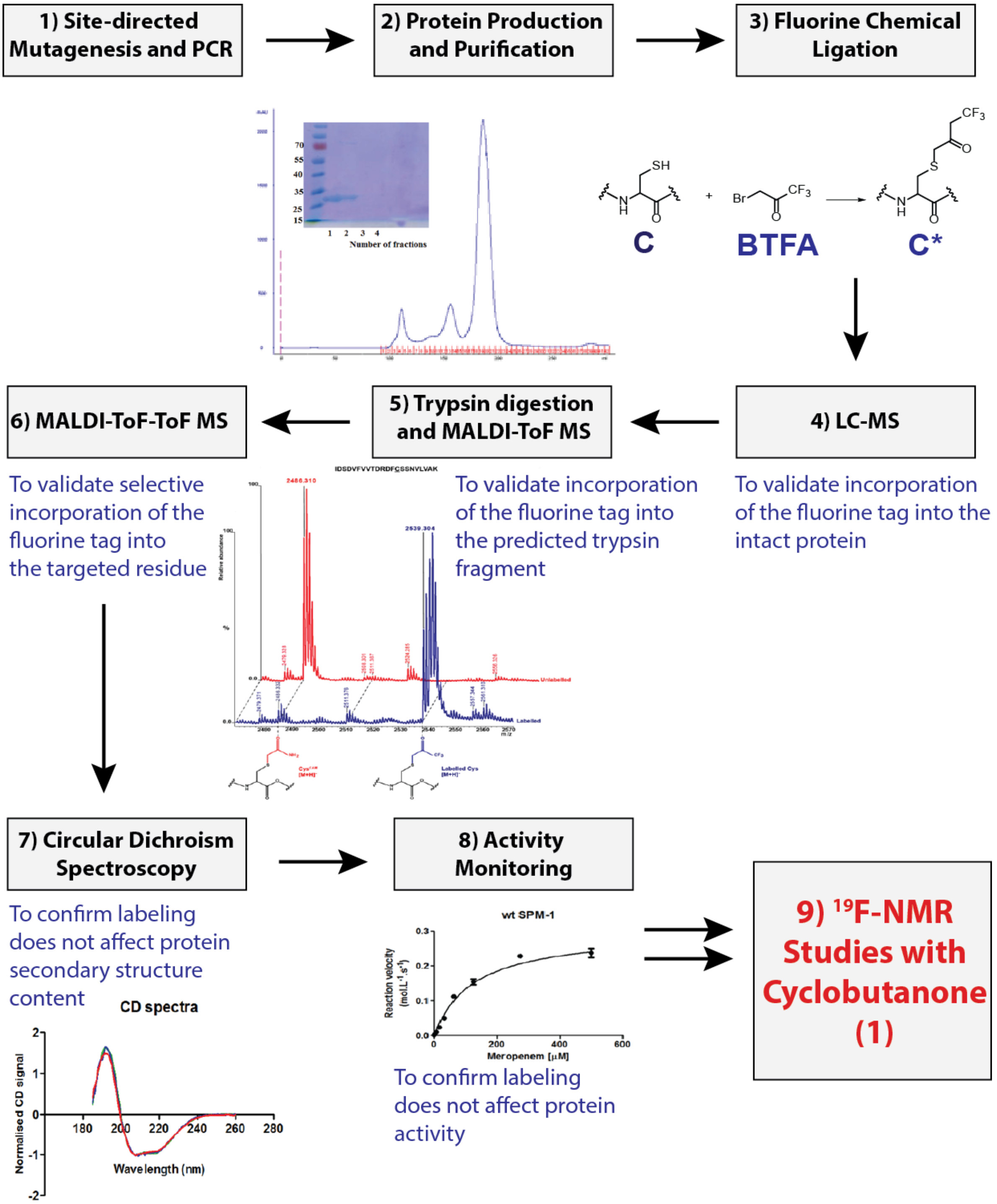

***Figure S4. Outline scheme of the labeling method and labeling validation steps.*[2a]** Recombinant SPM-1 variants (Y58C and Y152C) were generated by site-directed mutagenesis and produced following a three-step based purification method as reported . LC-MS analyses verified the masses of the recombinant proteins which were in agreement with the calculated masses. The observed mass difference between the unlabeled protein and its labeled counterpart corresponded to the attachment of a single CH_2COCF_3-label per ^{19}F-labeled SPM-1 positive ion. The specificity of the labeling method was evaluated by MALDI-ToF-ToF analyses following tryptic digestion. CD analyses and activity monitoring followed to confirm that labeling did not affect the protein secondary structure content and activity, respectively.[2a] SPM-1* variants were then used to study the binding of **1**.

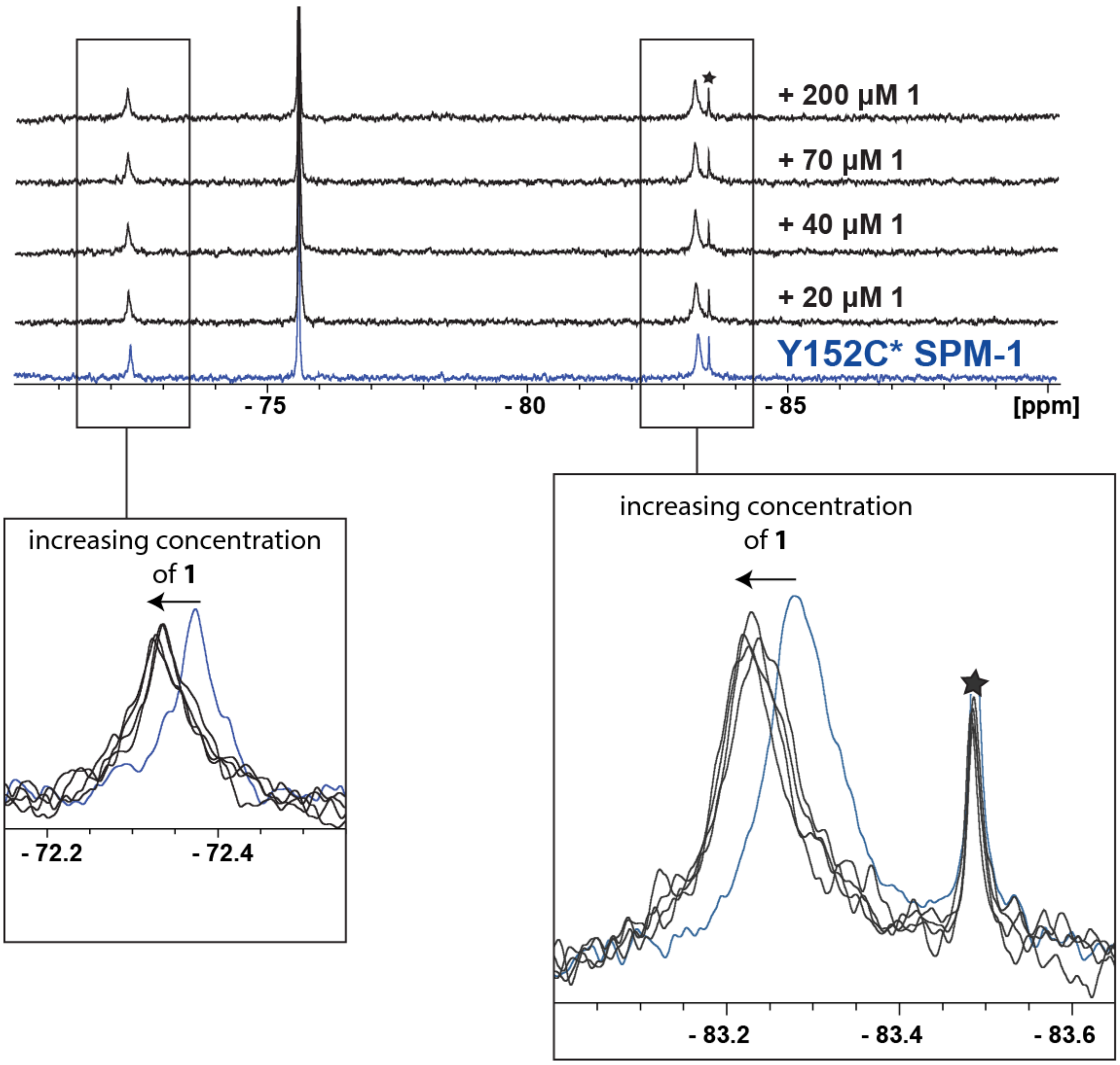

Figure S5. ***^{19}F NMR spectra of Y152C* SPM-1 (lower trace, blue) and Y152C* SPM-1 with increasing concentration of cyclobutanone 1.*** The assay mixture contained the Y152C* di-Zn(II)-SPM-1* variant in Tris buffer (50 mM, pH 7.5) supplemented with 200 mM NaCl and 10% (v/v) D_2O. Trifluoroacetic acid (50 µM) was used as an internal NMR standard (-75.45 ppm). The star denotes the peak corresponding to residual labeling reagent (3-bromo-1,1,1-trifluoroacetone). The peaks corresponding to the assigned closed (-83.2796 ppm) and open (-72.3741 ppm)[2b] conformations are highlighted in boxes and overlaid with the traces corresponding to increasing concentration of the cyclobutanone analogue **1**.

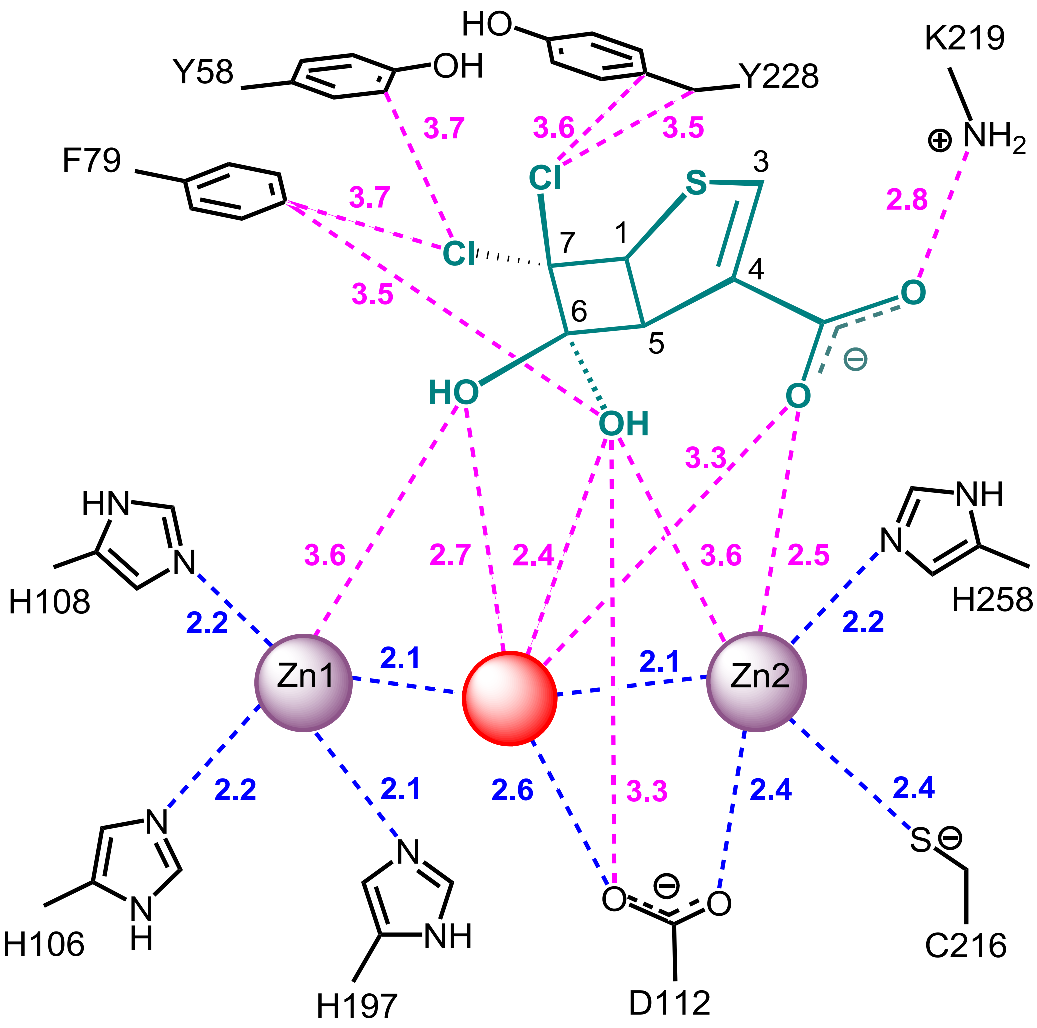

Figure S6. Interactions of cyclobutanone 1 with SPM-1 active site as observed in chain B. Crystallographically observed distances between the cyclobutanone inhibitor **1** (blue) and active site in chain B are shown in magenta (top panel). Distances between active site atoms are shown in blue (PDB ID: 5NDB). The red sphere represents the bridging water molecule or hydroxide. Numbers indicate distances in Å. See main text for chain A.

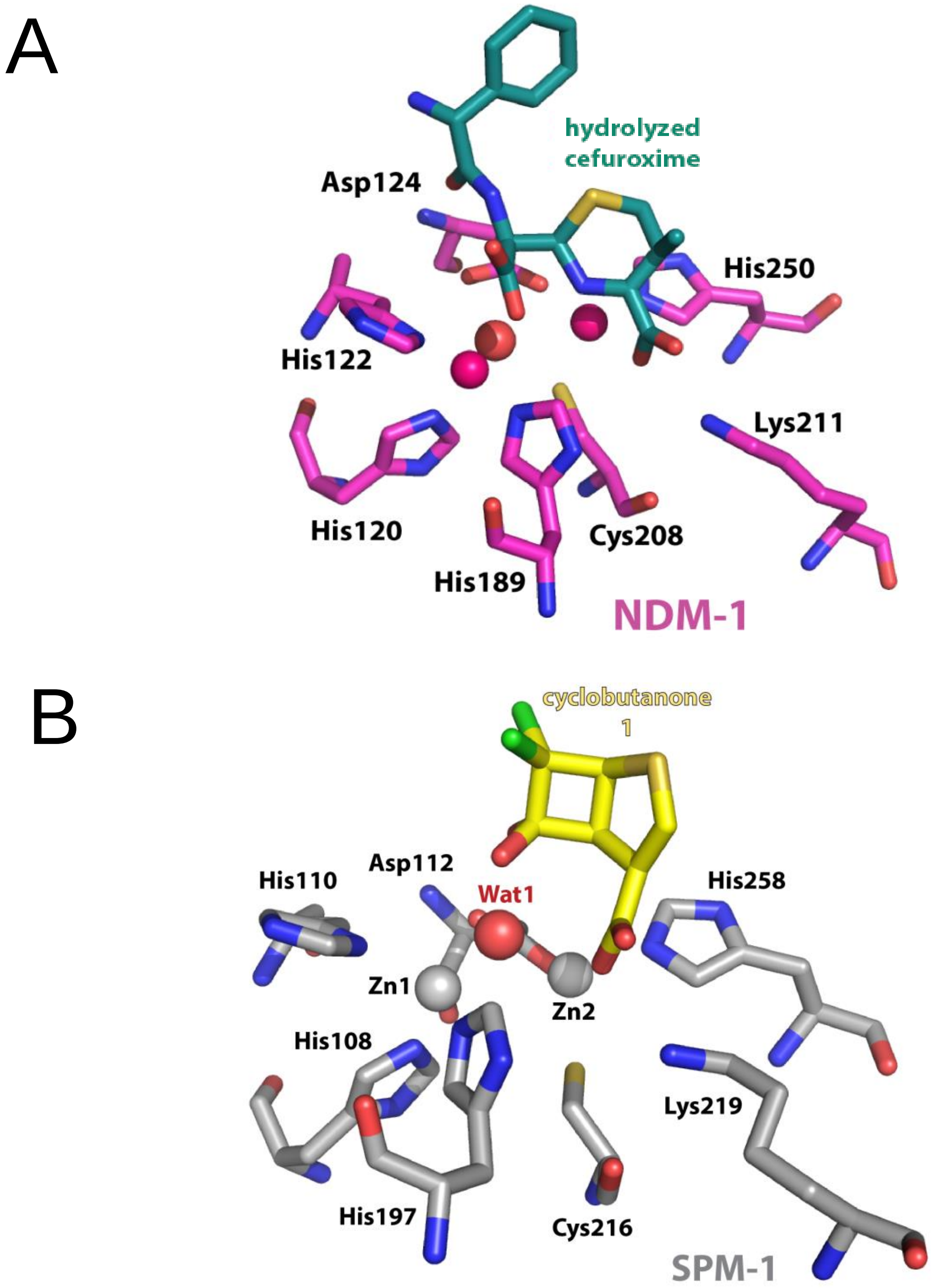

Figure S7. Comparative views from crystal structures of (A) hydrolyzed cefuroxime binding to NDM-1, a B1 MBL, (PDB ID: 4RL2)[13] and of (B) cyclobutanone 1 binding to SPM-1. The C4 carboxylate of cyclobutanone **1** is positioned to interact with both Zn2 (2.48 Å distance) and Lys219 (2.91 Å), a binding mode likely involved in substrate carboxylate binding in B1 MBLs.[13] The interaction of **1** with Zn2 is proposed to mimic the coordination of the cephalosporin-derived dihydrothiazine ring in the anionic intermediate.

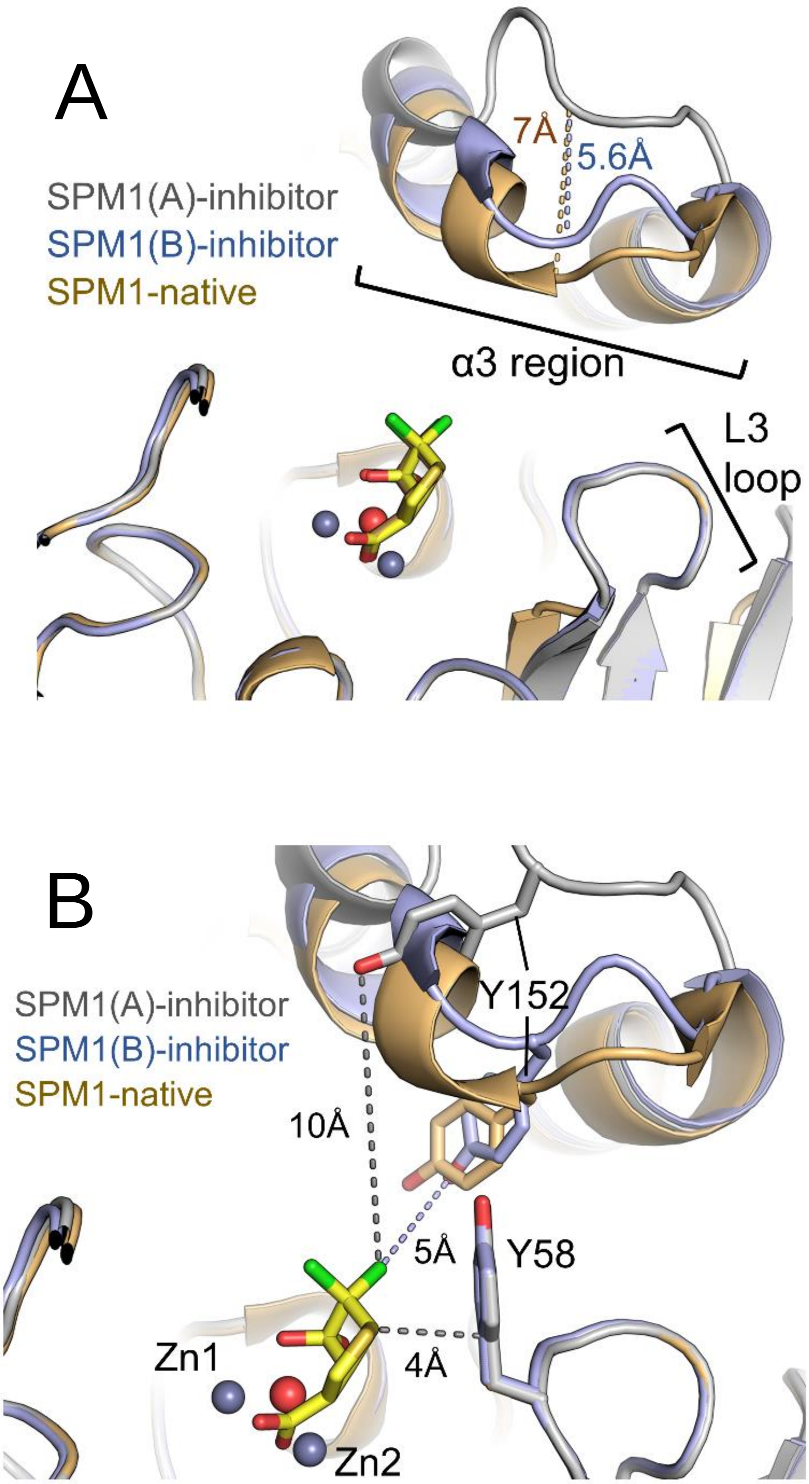

Figure S8. Conformations of SPM-1 in cyclobutanone-bound crystal structures. (A) Superposition of non-complexed SPM-1 (orange) with chain A (grey) and B (blue) of the SPM-1:cyclobutanone complex. While there is no observed conformational change in the L3 loop on inhibitor binding, the α3 region is relatively flexible (B-factors of 75 $Å^2$, chain A and 64 $Å^2$, chain B), and undergoes a substantial 7 Å shift away from the active site. (B) The positions and movement of Tyr58 and Y152 in unliganded and inhibitor-bound SPM-1. In the crystal structure, Tyr58 is ~4 Å away from bound cyclobutanone, while movement in the α3 region places Y152 5 Å (chain B) or 10 Å (chain A) from the cyclobutanone.

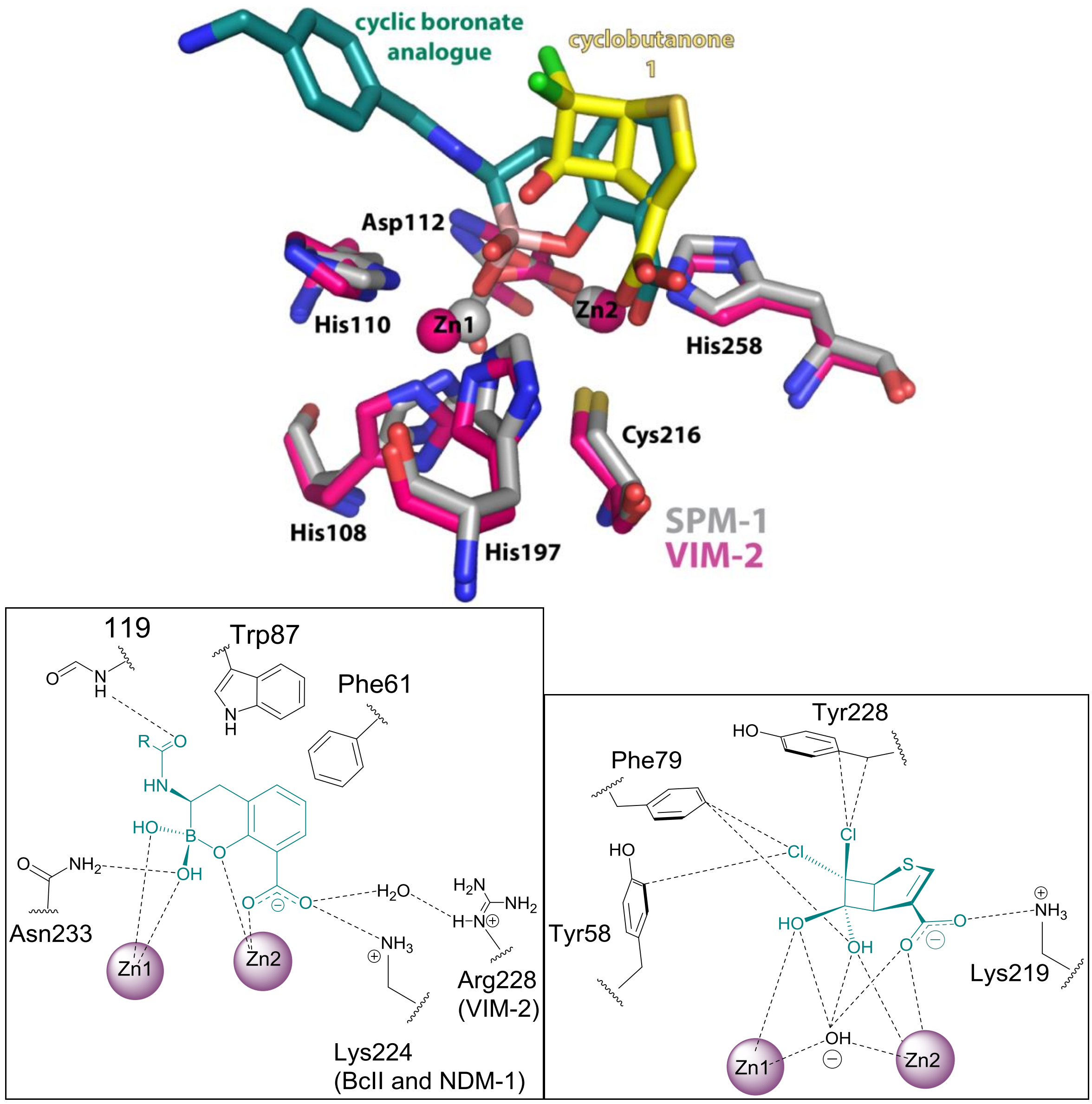

Figure S9. Comparative overlaid views from crystal structures of a boronate inhibitor binding to the B1 MBL VIM-2 (PDB ID: 5FQC)[14] and of cyclobutanone 1 binding to SPM-1. Binding of the boronate is proposed to mimic that of bicyclic β-lactams.[14] Structures were superimposed using PyMOL. One oxygen atom of the boronate C-3 carboxylate coordinates to Zn2; the other carboxylate oxygen interacts with Lys224 (NDM-1 and BcII) or Arg228 (*via* a bridging water molecule in VIM-2) by hydrogen-bonding/electrostatic interactions. These interactions are analogous to those with cyclobutanone **1** where the C-4 carboxylate binds Zn2 and Lys219 (similar to hydrolyzed cefuroxime, **Figure S7**).

Supplementary Tables

Table S1: Data Collection and Refinement Statistics

MβL	SPM-1	SPM-1
Inhibitor	cyclobutanone	native
**Data collection**		
Beamline	DLS I03	DLS I03
Space group	$P4_222$	$P4_222$
Molecules/ASU	2	2
Cell dimensions		
a, *b*, *c* (Å)	130.76, 130.76, 102.76	130.74, 130.74, 100.86
a, b, g (°)	90.0, 90.0, 90.0	90.0, 90.0, 90.0
Wavelength	0.92	0.92
Resolution (Å)	29.63 – 2.38 (2.48 – 2.38)	36.26 – 1.70 (1.94 – 1.70)
R_{pim}	0.048 (0.411)	0.029 (0.477)
CC 1/2	0.998 (0.833)	1.0 (0.690)
$I / \sigma I$	15.6 (2.7)	21.3 (2.4)
Completeness (%)	99.1 (92.5)	100.0 (100.0)
Redundancy	8.5 (8.3)	20.8 (21.3)
**Refinement**		
Resolution (Å)	29.63 – 2.38	36.26 – 1.70
No. reflections	35920	96042
R_{work} / R_{free}	0.1795 / 0.2178	0.1619 / 0.1725
No. atoms		
Protein	3868	3886
Solvent/Ions	286	563
Inhibitor	28	-
B-factors		
Protein	47.2	31.7
Solvent/Ions	55.4	45.1
Ligand	76.6	-
R.m.s. deviations		
Bond lengths (Å)	0.008	0.007
Bond angles (°)	1.132	1.079

*Values in parentheses are for highest-resolution shell.

References

[1] a) J. W. Johnson, D. P. Evanoff, M. E. Savard, G. Lange, T. R. Ramadhar, A. Assoud, N. J. Taylor, G. I. Dmitrienko, *J Org Chem* **2008**, *73*, 6970-6982; b) J. W. Johnson, M. Gretes, V. J. Goodfellow, L. Marrone, M. L. Heynen, N. C. Strynadka, G. I. Dmitrienko, *J Amer Chem Soc* **2010**, *132*, 2558-2560.

[2] a) M. I. Abboud, P. Hinchliffe, J. Brem, R. Macsics, I. Pfeffer, A. Makena, K. D. Umland, A. M. Rydzik, G. B. Li, J. Spencer, T. D. Claridge, C. J. Schofield, *Angew Chem Int Ed Engl* **2017**; b) J. Brem, W. B. Struwe, A. M. Rydzik, H. Tarhonskaya, I. Pfeffer, E. Flashman, S. S. van Berkel, J. Spencer, T. D. Claridge, M. A. McDonough, J. L. Benesch, C. J. Schofield, *Chem Sci* **2015**, *6*, 956-963.

[3] W. Kabsch, *Acta Crystallogr D Biol Crystallogr D* **2010**, *66*, 125-132.

[4] T. G. G. Battye, L. Kontogiannis, O. Johnson, H. R. Powell, A. G. W. Leslie, *Acta Crystallogr D Biol Crystallogr* **2011**, *67*, 271-281.

[5] M. D. Winn, C. C. Ballard, K. D. Cowtan, E. J. Dodson, P. Emsley, P. R. Evans, R. M. Keegan, E. B. Krissinel, A. G. W. Leslie, A. McCoy, S. J. McNicholas, G. N. Murshudov, N. S. Pannu, E. A. Potterton, H. R. Powell, R. J. Read, A. Vagin, K. S. Wilson, *Acta Crystallogr D Biol Crystallogr* **2011**, *67*, 235-242.

[6] A. J. McCoy, R. W. Grosse-Kunstleve, P. D. Adams, M. D. Winn, L. C. Storoni, R. J. Read, *J Appl Crystallogr* **2007**, *40*, 658-674.

[7] P. Emsley, K. Cowtan, *Acta Crystallogr D Biol Crystallogr* **2004**, *60*, 2126-2132.

[8] P. D. Adams, P. V. Afonine, G. Bunkoczi, V. B. Chen, I. W. Davis, N. Echols, J. J. Headd, L.-W. Hung, G. J. Kapral, R. W. Grosse-Kunstleve, A. J. McCoy, N. W. Moriarty, R. Oeffner, R. J. Read, D. C. Richardson, J. S. Richardson, T. C. Terwilliger, P. H. Zwart, *Acta Crystallogr D Biol Crystallogr* **2010**, *66*, 213-221.

[9] V. B. Chen, W. B. Arendall, J. J. Headd, D. A. Keedy, R. M. Immormino, G. J. Kapral, L. W. Murray, J. S. Richardson, D. C. Richardson, *Acta Crystallogr D Biol Crystallogr* **2010**, *66*, 12-21.

[10] M. I. Page, A. Badarau, *Bioinorg Chem Appl* **2008**, *2008*.

[11] J. H. Toney, G. G. Hammond, P. M. Fitzgerald, N. Sharma, J. M. Balkovec, G. P. Rouen, S. H. Olson, M. L. Hammond, M. L. Greenlee, Y. D. Gao, *J Biol Chem* **2001**, *276*, 31913-31918.

[12] G. Garau, C. Bebrone, C. Anne, M. Galleni, J. M. Frere, O. Dideberg, *J Mol Biol* **2005**, *345*, 785-795.

[13] H. Feng, J. Ding, D. Zhu, X. Liu, X. Xu, Y. Zhang, S. Zang, D. C. Wang, W. Liu, *J Am Chem Soc* **2014**, *136*, 14694-14697.

[14] J. Brem, R. Cain, S. Cahill, M. A. McDonough, I. J. Clifton, J. C. Jimenez-Castellanos, M. B. Avison, J. Spencer, C. W. Fishwick, C. J. Schofield, *Nat Commun* **2016**, *7*, 12406.
